# Exosomal PSM-E inhibits macrophage M2 polarization to suppress prostate cancer metastasis through the RACK1 signaling axis

**DOI:** 10.1186/s40364-024-00685-8

**Published:** 2024-11-14

**Authors:** Xingliang Qin, Ruoxi Niu, Yongyao Tan, Yuxin Huang, Weishu Ren, Weiwei Zhou, Huiquan Wu, Junlong Zhang, Mingze Xu, Xiang Zhou, Hongyu Guan, Xun Zhu, Yu Chen, Kaiyuan Cao

**Affiliations:** 1 Department of Endocrinology, The First Affiliated Hospital of Sun Yat-sen University, Guangzhou, 510080 China; 2 School of Biomedical Sciences, The Chinese University of Hong Kong, Hong Kong, 999077 China; 3 Key Laboratory of Tropical Disease Control (Sun Yat-sen University), Ministry of Education, Guangzhou, 510080 China; 4 Research Center for Clinical Laboratory Standard, Department of Immunology and Microbiology, Zhongshan School of Medicine, Sun Yat-sen University, 74 Zhongshan Road II, Guangzhou, 510080 China; 5 Department of Urology, The First Affiliated Hospital of Sun Yat-sen University, Guangzhou, 510080 China; 6 Guangzhou Jishiyuan Bio-technology Co., Ltd., Guangzhou, 510700 China; 7 Department of Microsurgery, Trauma and Hand Surgery, The First Affiliated Hospital, Sun Yat-sen University, Guangzhou, 510080 China

**Keywords:** Prostate cancer, Exosome, PSM-E, Macrophage polarization

## Abstract

**Background:**

It is well-established that understanding the mechanism of prostate cancer (PCa)-associated metastasis is paramount for improving its prognosis. Metastasis is known to involve the communication between tumor-associated macrophages (TAMs) and tumor cells. Exosomes are crucial in mediating this intercellular communication within the tumor microenvironment. Nonetheless, the role of exosomal proteins in PCa metastasis is not yet fully understood. Here, we investigated the mechanisms of prostate cancer-derived exosomal PSM-E on regulating macrophage M2 polarization to suppress tumor invasion and metastasis.

**Methods:**

PSM-E levels in exosomes were detected by transmission electron microscopy and Western blotting analysis. The diagnostic value of urine-derived exosomal PSM-E in PCa were evaluated by LC-MS/MS, correlation analysis, and ROC curves analysis. The mechanisms underlying the inhibitory effect of exosomal PSM-E on the M2 polarization of macrophages was investigated by co-IP, IHC staining, and PCa tumorigenesis model, etc.

**Results:**

We revealed that exosomal PSM-E is upregulated in exosomes derived from the serum and urine of PCa patients. Clinically, an elevated exosomal PSM-E expression in urine is significantly correlated with an advanced pathological tumor stage and a high Gleason score. Our research also revealed that exosomal PSM-E inhibits prostate cancer cell proliferation, invasion, and metastasis by suppressing macrophage polarization in vitro and in vivo. Furthermore, we provided compelling evidence that exosomal PSM-E inhibits M2 polarization of macrophages by recruiting RACK1 and suppressing the FAK and ERK signaling pathways, consequently suppressing PCa invasion and metastasis. Furthermore, we found that the protease-associated domain of PSM-E and the fourth tryptophan-aspartate repeat of RACK1 are crucial for the interaction between PSM-E and RACK1.

**Conclusions:**

Notably, exosomes carrying PSM-E from PCa urine could potentially serve as a biomarker for PCa, and targeting exosomal PSM-E may represent a strategy for preventing tumor progression in this patient population.

**Supplementary Information:**

The online version contains supplementary material available at 10.1186/s40364-024-00685-8.

## Background

Prostate cancer (PCa) is a prevalent cancer and the second leading cause of cancer-related death among men, making it a growing global health concern [1, 2]. While early-stage PCa is treatable, a third of cases progress to a more aggressive form associated with poor survival [1, 2]. Despite the advent of various therapeutic strategies, such as androgen deprivation therapy (ADT), tackling metastases remains challenging. Therefore, there is a pressing need for a deeper understanding of PCa metastasis to develop effective treatments [3]. While prostate-specific antigen (PSA) is frequently employed as a biomarker for PCa diagnosis and prognosis, clinically significant PCa cannot be consistently differentiated from prostatitis or benign prostatic hyperplasia (BPH) due to limited sensitivity and specificity of PSA detection, which leading to over diagnosis and overtreatment [4, 5]. Hence, there is an urgent need to identify more efficient biomarkers and develop more sensitive approaches for early diagnosis of PCa.

There is an increasing consensus suggesting that macrophages in a tumor microenvironment, tumor-associated macrophages (TAMs), are considered a significant contributor to PCa progression [6, 7]. Of note, Macrophages come in two distinct polarized forms: M1, which enhances immune responses and acts against tumors, and M2, which suppresses immune activities and promotes tumorigenesis [6–8]. Emerging evidence suggests that TAMs in tumors and nearby tissues, resembling M2 macrophages, exhibit pro-tumor characteristics, such as promoting PCa cell growth, invasion, and metastasis [6–8]. Furthermore, TAM infiltration is an independent risk factor for PCa recurrence, by reason of patients with fewer TAMs tend to have a better prognosis [9, 10]. In this respect, it has been reported that the prognosis of PCa patients with decreased TAM abundance is significantly better than those with higher abundance, indicating its relationship with the prognosis of PCa [9, 10]. Accumulating evidence has indicated TAM, characterized by their abundance and active infiltration within the tumor microenvironment, play a crucial role in other malignancies, such as colorectal cancer [11], lung cancer [12] et al.. The initiation and progression of tumors represent a complex process involving genetic alterations in cancer cells as well as multiple factors derived from the surrounding tumor cell microenvironment.

Growing evidence suggests that tumor-released exosomes that are a kind of extracellular vesicles subpopulations with diameters ranging from 30 to 150 nm, can act as a carrier for intercellular signal exchange and are vital in shaping the tumor microenvironment [13–15]. These exosomes carry cargos like mRNA, proteins, and non-coding RNA, influencing the recipient cells and their microenvironment [13–15]. It has been established that tumor cells exosome-delivered proteins can regulate manipulate immune cells and establish a microenvironment conducive to tumor growth and metastasis [13–15]. Exosomes are integral in prostate cell-cell communication, modulating the functions of recipient cells, and potentially alerting the tumor microenvironment, which may subsequently influence the progression of PCa. Numerous studies have documented the selective packaging of proteins into exosomes, facilitating various prostate cellular and biological functions. And the protein composition of exosomes is contingent upon the specific cell types and biological fluids from which they are derived [16]. It has been demonstrated by Urabe et al.. that extracellular vesicles derived from metastatic prostate cancer can facilitate osteoclastogenesis by transferring CUB-domain containing protein 1 (CDCP) molecules [17]. Lu et al. revealed that tumor cell exosomes could regulate monocyte M2 polarization through integration of α_v_β_6_ integrin by cycling intracellularly in PCa progression [18]. Yingying Shen et al.. reported that tumor-derived exosomes modulate dendritic cell function to facilitate tumor metastasis through the HSP72/HSP105-TLR2/TLR4 signaling pathway, which suggesting that HSP72 and HSP105 present on serum exosomes may serve as potential markers for predicting tumor metastasis [19]. Hosseini-Beheshti et al. identified several constitutive proteins as novel biomarkers or therapeutic targets, which were enriched in exosomes derived from six PCa cells with distinct androgen receptor phenotypes by proteomics profiling [20]. However, the involvement of tumor-derived exosomes and TAMs in PCa progression warrants further investigation.

Prostate-specific membrane antigen (PSMA), primarily produced by prostate epithelial cells, is a type II transmembrane glycoprotein with functions related to N-acetylated α-linked acidic dipeptidase and folate hydrolase. It is significantly elevated in conditions like BPH, PCa, castration-resistant PCa, and metastatic PCa, and its expression levels correlate with tumor grade and stage [21, 22]. Notably, our group found that a novel identified alternatively spliced PSMA variant, namely PSM-E, is also specifically overexpressed in PCa and correlated strongly with PCa stage and grade [23]. Subsequently, our study has shown that PSM-E can inhibit the proliferation, migration, and invasiveness of PCa cells [23, 24]. Current evidences indicate that PSMA-containing exosomes hold promise as a target for early PCa diagnosis, treatment response assessment, and prognosis [25–27]. Recently, we proposed that PSM-E could suppress the production of inflammatory cytokines in the tumor microenvironment and inhibit monocyte chemotaxis [28]. Importantly, our findings further revealed PSM-E expression was negatively correlated with the infiltration of M2-like TAMs in PCa tissue [28]. PSM-E holds significant potential for early diagnosis and treatment response of PCa assessment owing to its high tissue and pathological specificity. Despite these discoveries, the mechanisms associating PSM-E with the tumor microenvironment in PCa progression remain unclear, warranting further research.

This work focused on the impact of PSM-E in the tumor microenvironment on PCa progression and the underlying mechanisms. We provided hitherto undocumented evidence of PSM-E-containing exosomes in the extracellular environment of PCa patients, including serum and urine samples. Correlation analysis revealed a close association between the expression of urine exosomal PSM-E and the clinical stage and Gleason score in PCa patients. We also identified the ability of cellular exosomes to deliver PSM-E protein intercellularly, thereby transmitting its tumor-inhibiting and anti-metastatic effects. Furthermore, we revealed the involvement of PSM-E in macrophage polarization, suggesting that PCa-derived PSM-E-containing exosomes contribute to the regulation of M2 macrophage differentiation, a key factor in PCa progression. Mechanistically, PSM-E-laden exosomes from PCa cells were proved to suppress the FAK and ERK signal pathways by binding with RACK1. Taken together, our study illustrates that PSM-E-containing exosomes can hinder the progression of PCa by influencing macrophage polarization and metastasis, making it a potential prognostic marker for PCa progression and a target for anti-PCa therapy.

## Methods

### Participants, blood, and urine sampling

Serum and urine samples were collected from PCa patients undergoing surgery at the Department of Urology, the Affiliated Hospital of Sun Yat-sen University. Clinicopathological diagnoses were confirmed by at least two pathologists following the American Joint Committee on Cancer (AJCC) guidelines. Inclusion criteria required patients to have undergone prostate needle biopsy and received a histological diagnosis of PCa. Neither had undergone neoadjuvant androgen deprivation therapy nor had they ever been diagnosed with cancer. The control group consisted of males with total PSA levels below 4 ng/ml, showing no significant changes in ultrasound imaging or signs of prostatitis. The study involved 45 control cases, and 48 PCa cases. The first morning urine samples (10 ml) were collected from all participants, and peripheral venous blood was drawn into sodium citrate-free blood collection tubes to obtain serum. Both urine and blood samples were taken prior to any treatment. Prior to exosome was extracted from serum and urine samples of PCa patients by centrifugation of serum and urine at 3000 rpm for 10 min to remove cellular debris and deposits. This study followed ethical principles approved by the Research Ethics Committee of the First Affiliated Hospital of Sun Yat-sen University.

### Cell culture

Human PCa cell lines (PC3 and LNCaP) and human THP-1 were cultured in RPMI-1640 (Invitrogen, Carlsbad, CA), and the human kidney cell line 293T was cultured in DMEM (Invitrogen), respectively. The culture medium was supplemented with 10% (v/v) fetal bovine serum (FBS) (Invitrogen), 2 mM L-glutamine, 2 mM nonessential amino acids, 100 µg/ml streptomycin, and 100 units/ml penicillin (Invitrogen). Cells were maintained in an incubator under 37 °C and a humidified atmosphere with 5% CO_2_. THP-1 cells (1 × 10^6^ cells per well) were seeded in 60 mm dishes and settled for 24 h before being treating with 100 nM phorbol myristate acetate (PMA, Spring & Autumn, Nanjing, China), followed by culture with 20 ng/ml IL-4 for an additional 48 h to differentiate into M2 macrophages.

### Plasmids and transfection

PSM-E plasmids with Flag epitope tags and RACK1 plasmids with HA epitope tags were amplified by RT-PCR and inserted into the pSin-EF2-puro vector. DNA sequencing was used to verify the plasmids. Small interfering RNA (siRNA) for PSM-E, purchased from Ruibo Biotechnology (Guangzhou, China). All PCR primers and siRNA sequence used in this work is shown in Supplementary Table S1. Plasmids and siRNA were transfected at specified concentrations using Lipofectamine 3000 reagents (Invitrogen) following the manufacturer’s guidelines. Lentivirus containing PSM-E segments was harvested from 293T cells that transfected with overexpressing plasmids (pSin-EF2-puro vector), with psPAX2 and pMD2G. 48 h after transfection, virus particles were harvested from the culture medium. PC3 and 293T cells were infected with lentivirus containing PSM-E ORF, and stable cell lines were selected and generated in DMEM supplemented with 5 µg/ml of puromycin.

### Isolation and characterization of exosome

Three distinct cell groups were utilized in this study, including 293T cells stably overexpressing PSM-E-Flag (referred to as 293T-PSM-E), PC3 cells stably overexpressing PSM-E-Flag (referred to as PC3-PSM-E), and LNCaP cells displaying high basal-level PSM-E protein. All these cell groups were grown to monolayer with 80–90% confluence in a medium supplemented with 10% of exosome-free FBS. Then, the conditioned medium was harvested and centrifuged twice (3000 × g for 15 min) to remove cells and centrifuged once (20,000 × g for 30 min) to remove cellular debris. Subsequently, the supernatants were further centrifuged at 100,000 × g for 70 min, and all these steps were meticulously performed at 4 °C [29]. Then, the supernatants were concentrated ten-fold using an Amicon Ultra-15 centrifugal filter unit with a 3 kDa cutoff value. Exosomes derived from serum and urine were isolated using an Exosome Concentration Isolation Kit (Liaoning Rengen Biosciences Co., Ltd, Liaoning, China). These purified exosomes were resuspended in PBS. The protein content of these exosomes was quantified using the Bicinchoninic Acid protein assay kit (BCA, Thermo Scientific Pierce, Rockford, IL) with BSA as a reference, following the manufacturer’s guidelines. As described previously, the exosomes were visualized through negative staining and analyzed via transmission electron microscopy (TEM) [30]. Finally, using Western blotting analysis, we characterized the exosomes loaded with PSM-E and excised the molecular weight-specific band from the gel for subsequent mass spectrometry analysis (MS).

### Western blotting analysis

Cells were lysed using lysis buffer (composed of 50 mmol/L Tris-HCl with pH 7.4, 6% SDS, 5% mercaptoethanol, 10% glycerol, 1 mmol/L PMSF, and 0.1% bromophenol blue). The protein concentration of the lysates was quantified using the BCA assay kit with BSA as a reference, per the instructions. Subsequently, the lysed proteins were separated through SDS-PAGE and transferred onto PVDF membranes (Millipore, Billerica, MA). Various primary antibodies were used, such as anti-CD63 (Santa Cruz Biotechnology, Dallas, Texas), anti-CD206 (Abcam, Cambridge, UK), anti-Flotillin-2 (Abcam), anti-Flag (Cell Signaling Technology, Beverly, MA), anti-RACK1 (Abcam), anti-HA (CST), anti-ERK (CST), anti-p-ERK (Cell Signaling Technology), anti-FAK (CST), anti-p-FAK, and anti-GAPDH (ABclonal Technology, Wuhan, China). Incubation with these antibodies was carried out overnight at 4 °C, followed by a 1-hour incubation with anti-rabbit (ABclonal Technology) or anti-mouse (ABclonal Technology) secondary antibodies at room temperature. GAPDH was employed as a loading control for normalization in quantification. An enhanced ECL reagent (Thermo Fisher Scientific, Rockford, IL) was used to visualize the chemiluminescence bands.

### LC-MS/MS analysis of PSM-E

The study utilized the Waters Series ACQUITY Premier system (Waters Corporation, Milford) and a Xevo TQ-S LC/MS mass spectrometer (Waters Corporation, MA). Liquid chromatography separations were carried out on a ZORBAX Eclipse XDB-C18 (Agilent, Waldbronn, Germany) at room temperature. The mobile phase comprised solvent A (0.1% formic acid in water) and solvent B (0.1% formic acid in methanol). A linear gradient with a flow rate of 0.3 mL/min was applied in the following manner: 10% B (0 min), 10% B (1 min), 90% B (4 min), 90% B (8 min), and 10% B (9 min). An electrospray ion source was used to interface the mass spectrometer with the positive MRM mode. The injection volume was 10 µl. The electrospray capillary voltage was optimized to 70 V, and the nebulizer pressure was set to 35 psi. The data were collected using the Waters MassLynx Mass Spectrometry Software (version 4.1).

### Wound healing assay

Cells (1 × 10^6^ cells/well) were seeded in a six-well plate, ensuring they reached 90% confluence at the time of scratch wounding. To inhibit cell mitosis, cells were pretreated with mitomycin C at 10 µg/ml (Sigma-Aldrich) for 2 h. The non-adherent cells were eliminated by washing them with a culture medium after three straight scratch wounds were created in each well using a pipette tip (10 µl). Purified exosomes derived from 293T-PSM-E cells or 293T-Vector cells were added to the wells and incubated for up to 72 h. Using a bright-field microscope, the wound width was measured immediately after wounding (at 0 h) and at 24 h.

### Invasion assays

The invasion assays were conducted using cell culture inserts with 8.0 μm pore size (Falcon, Corning, Tewksbury MA), coated with 10% Matrigel or not (BD Biosciences). In brief, the upper chamber was seeded with PC3, and the lower chamber was incubated overnight with 500 µl of fresh medium. The medium was then replaced with fresh medium, and the cells were incubated with medium alone or stimulated with purified exosomes derived from 293T-PSM-E cells or 293T-Vector cells for 72 h. Those cells in the upper chamber that were non-migratory were removed, and the remaining cells were gently washed with 1×PBS. A 0.5% crystal violet solution was used to stain these adhered cells that had migrated through the filter for 10 min. Images were captured using light microscopy.

### RNA extraction and quantitative real-time PCR

Total RNA was extracted using TRIzol (Invitrogen, Carlsbad, CA) following the manufacturer’s instructions. The first-strand cDNA was synthesized using HiScript II Q Select RT SuperMix for qPCR (Vazyme, Jing Nan, China), and real-time PCR was performed using ChamQ SYBR qPCR Master Mix (Vazyme) or AceQ qPCR Probe Master Mix (Vazyme) according to the manufacturer’s protocols. The primer groups used for real-time PCR are provided in Supplementary Table 1. Three independent experiments were conducted, and the data were expressed as ratios relative to GAPDH and presented as the mean ± S.D. values.

### Co-immunoprecipitation

Cells transfected with the specified plasmids for 48 h were harvested and collected with lysis buffer (1 mmol/L EDTA, 25 mmol/L HEPES, 2% glycerol, 150 mmol/L NaCl, 1% NP-40, and cocktail (Roche, Basel, Switzerland). Mixed lysates were kept on ice for 30 min, followed by 10 min of centrifugation at 12,000 rpm. For pre-cleaning, the supernatant was incubated with 20 µl of agarose beads (Calbiochem) for 1 h with rotation at 4 °C. After centrifuging at 2,000 rpm for 1 min, the lysates were then incubated overnight at 4 °C with the indicated antibodies or IgG antibody. Then, the precipitates were washed three times with IP wash buffer (150 mmol/L NaCl, 20 mmol/L HEPES, 2% glycerol, 1 mmol/L EDTA, 0.1% NP-40, and 1 mmol/L EGTA). After completely removing the liquid, the bound proteins were resolved in 1× sample buffer, subjected to SDS-PAGE, and detected by Western blotting analysis.

### In vivo tumorigenesis model

C57BL/6J mice aged 5–6 weeks were obtained from Guangzhou Forevergen Medical Laboratory Animal Center and housed in specific pathogen-free (SPF) barrier facilities on a 12-hour light/dark cycle. On day zero, RM-1 cells (1 × 10^5^) were subcutaneously injected into the right dorsum. Before treatment, mice were randomly divided into two treatment groups and two control groups in a blinded fashion, with each group consisting of five mice. On day 7, when tumors had formed and were measurable, two treatment groups of mice received intraperitoneal (*i.p.*) injections of PC3-Vector-Exosomes (10 µg/kg body weight) and LNCaP-siPSM-E-Exosomes (10 µg/kg body weight) every three days. In contrast, the vehicle-control animals received *i.p.* doses of 200 µl PC3-Vector-Exosomes or LNCaP-siNC-Exosomes solution per mouse daily. Every four days, the diameter of each mouse tumor was measured with a digital caliper by measuring perpendicular diameters in a blinded manner. And tumor volume was calculated using the formula: tumor volume (mm^3^) = (length [mm]) × (width [mm])^2^ × 0.5. The weight of each mouse tumor was also recorded. We euthanized all animals, and dissected and weighed the tumors at the end of the experiment. All procedures involving animals were approved by the Ethics Committee of Guangzhou Forevergen Medical Laboratory Animal Center under approval No. IACUC-AEWC-F2305037.

### Immunohistochemical staining (IHC)

IHC analysis was performed in accordance with the protocol previously described [31]. In brief, Paraffin sections of mouse prostate cancer tissues were prepared and firstly deparaffinized and hydrated. Microwave method used to retrieve antigens in the, followed by incubation with 0.3% hydrogen peroxide solution to block endogenous peroxidase activity. An anti-CD206 antibody (CST) was used to detect CD206 level in mouse tumors tissue. An antibody against Ki67 (Servicebio; Wuhan, China) were used to detect Ki67 level in mouse tumors tissue. The sections were developed using 3,3’-diaminobenzidine tetrahydrochloride and peroxidase after incubation with primary and secondary antibodies. After counterstaining with hematoxylin, the sections were mounted in a nonaqueous mounting medium. Images were visualized using a Leica DM2500 microscope system (Leica, Germany). Image J Version 1.5i software (Media Cyernetics, Inc, USA) was used to evaluate the percentage of the staining positively area.

### Statistical analysis

This study was carried out using the GraphPad Prism software (version 9.5) for all statistical analyses. Data conforming to normal distribution were presented as means ± standard deviations (S.D.) from three independent experiments. Comparisons between the two groups were made using independent Student’s *t*-tests. The one-way ANOVA was used for multiple comparisons of continuous variables, followed by the Tukey-Kramer test. Data that non-normally distributed were expressed with the median and interquartile range, and the Kruskal-Wallis and Dunn’s tests were used for multiple comparisons. Correlations were determined through Spearman rank correlation analysis. Additionally, receiver-operating characteristic (ROC) analysis was carried out for the levels of PSM-E, tPSA, and f/t PSA in the context of PCa. Plotting true-positive fractions (sensitivity) against false-positive fractions (specificity) can be accomplished by changing the cutoff value used in the analysis. Diagnostic test accuracy is measured by the areas under ROC curves. Significant differences were denoted in charts as follows: *p < 0.05, **p < 0.01, and ***p < 0.001. *p* values less than 0.05 indicated a statistically significant difference, while “ns” indicated no significance. All statistical analyses were based on at least three biological replicates.

## Results

### Clinical characteristics of the study samples

A summary of the participants’ baseline characteristics is shown in Table 1, which encompassing age, Gleason score, TNM stage, lymph node metastasis, and PSA serum levels. Serum and urine samples were collected from 93 participants, including control (*n* = 45), and PCa (*n* = 48) cases.

**Table 1:** Associations between urine-derived exosomal PSM-E expression and clinicopathological characteristics of PCa patients

Characteristic	Cases (%) (*n* = 48)	PSM-E expression (%)		*p*
		High	Low	
Age				
< 66	16 (33.3)	8	8	0.838
≥ 66	32 (66.7)	17	15	
Gleason score				
≤ 6	6 (12.5)	2	4	0.028 *
3 + 4	12(25)	5	7	
4 + 3	11(22.9)	8	3	
≥ 8	19 (39.6)	10	9	
TNM stage				
I/II	17 (35)	5	12	0.003 **
III/IV	31 (65)	20	11	
Lymph node metastasis				
Yes	6 (12.5)	2	4	0.383
No	42 (87.5)	20	22	
fPSA				
< 4 ng/ml	40 (83.3)	19	21	0.113
≥ 4 ng/ml	8 (16.7)	6	2	

*p* value < 0.05 was considered significant. *p < 0.05, **p < 0.01

### PSM-E is encapsulated within serum and urine-derived exosomes from PCa patients

**Fig. 1:**
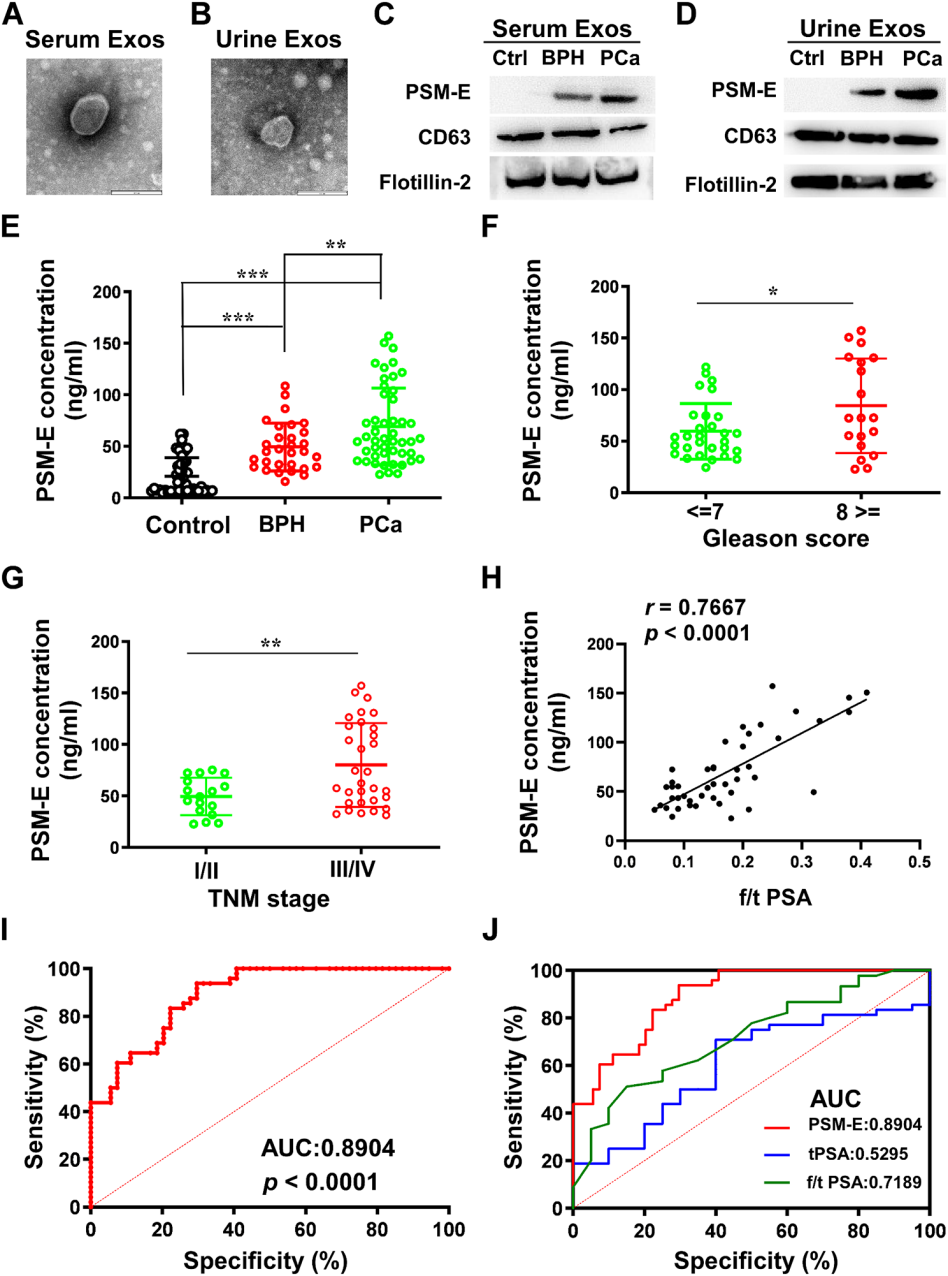
Clinical analysis of serum and urine-derived exosomal PSM-E in PCa. (**A**, **B**) Representative electron microscopy images of exosomes isolated from human serum and urine. (**C**, **D**) Exosomes were isolated from the serum or urine of healthy donors (control), BPH patients, and PCa patients, respectively. Purified exosomes derived from each group described above were analyzed by immunoblotting with anti-PSM-E, anti-CD63, and anti-Flotillin-2 antibodies. CD63 and Flotillin-2 served as the internal control of exosomes. (**E**) Urine-derived exosomal PSM-E was significantly higher in PCa patients than in the control group. (**F**, **G**) Urine-derived exosomal PSM-E was significantly higher in the high Gleason score and advanced pathological tumor stage than in the low Gleason score and pathological tumor stage. (**H**, **I, J**) Receiver operating characteristic (ROC) curves for detecting PSM-E, tPSA, or f/t PSA. Exos = exosomes. The data are presented as means ± SDs. *p < 0.05, **p < 0.01, and ***p < 0.001

Exosomes were isolated from serum or urine samples obtained from the participants using the corresponding commercial kits. Subsequently, exosomes purified from 5 samples underwent transmission electron microscopy and Western blotting analysis to detect PSM-E protein levels. The electron micrographs of exosomes revealed vesicle sizes primarily around 100 nm (Fig. 1A, B). Notably, PSM-E was enriched in serum-derived exosomes from BPH and PCa patients, and abundant in those isolated from urine samples of BPH and PCa patients (Fig. 1C, D). Importantly, PSM-E levels in PCa patients were significantly higher than those in control group. In contrast, exosomes from the control group did not contain detectable PSM-E, with CD63 and Flotillin-2 as exosomal control markers. In summary, these findings demonstrate that PSM-E is found in both the serum and urine of BPH and PCa patients.

### Urine-derived exosomal PSM-E yielded a good diagnostic performance for PCa

In an attempt to investigate the value of urine-derived exosomal PSM-E in PCa, LC-MS/MS analysis was used to detect the protein level of PSM-E in the exosomes of each group by recognizing the specific peptide FLAAYACTGCLAER of PSM-E. As shown in Fig. 1E, urine-derived exosomal PSM-E concentration was significantly elevated in the PCa patients compared to the control group (*p* < 0.001). Exosomal PSM-E expression exhibited significantly higher levels in patients with PCa than those with BPH (*p* < 0.01). It is documented that Gleason score is a critical grading system used to evaluate the aggressiveness of prostate cancer based on histological patterns observed in biopsy samples [5]. The score ranges from 2 to 10, with lower scores indicating less aggressive cancer and higher scores indicating more aggressive disease [5]. Moreover, prostate cancer is primarily classified using the tumor-node-metastasis (TNM) staging system, which is crucial for determining prognosis and treatment strategies [5]. Remarkably, high level of exosomal PSM-E positively correlated with a high Gleason score (≥ 8, *p* < 0.05) and advanced pathological tumor stage (III/IV TNM stage *p* < 0.01) (Fig. 1F, G,).

To analyze the diagnostic specificity and sensitivity of exosomal PSM-E for PCa compared with those of tPSA, ROC curves for the detection of PCa were applied. As shown in Fig. 1H, I, J in all PCa subjects, the values of AUC were 0.8904 (95% CI, 0.8311 to 0.9497) for exosomal PSM-E, 0.5295(0.3812 to 0.6779) for tPSA and 0.7189 (95% CI, 0.5890 to 0.8487) for f/t PSA. These differences between exosomal PSM-E and f/t PSA were statistically significant in all PCa subjects (*p* < 0.0001 and *p* < 0.001). Using a cutoff value of 42.08 ng/ml for exosomal PSM-E, exosomal PSM-E level significantly differentiated PCa patients from control cases among consecutive patients with 75% sensitivity and 80% specificity for diagnosing PCa. These results suggest that exosomal PSM-E exhibited good performance in distinguishing PCa and outperformed other traditional clinical biomarkers tPSA or f/t PSA.

### PSM-E can be exported from cells by exosomes

**Fig. 2:**
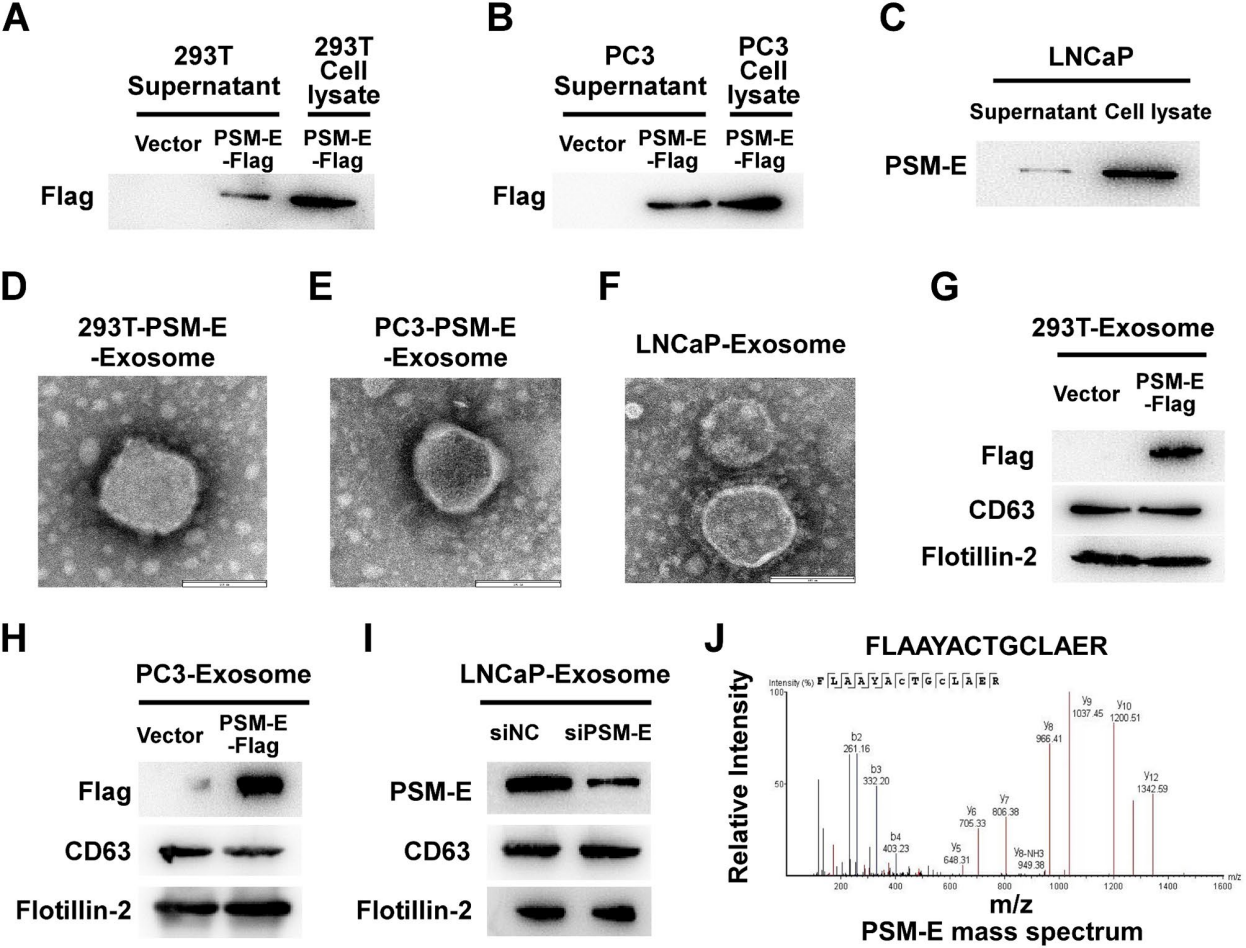
Identification of PSM-E-containing exosomes derived from 293T, PC3, and LNCaP cells. (**A**, **B**) Western blotting analysis of the expression levels of PSM-E in the supernatant and cell lysate of 293T or PC3 cells transfected with empty vector and PSM-E-Flag construct. (**C**) Western blotting analysis of the expression levels of PSM-E in the supernatant and cell lysate of LNCaP cells. (**D-F**) Representative electron microscope of exosomes isolated from the supernatant of PSM-E-transfected 293T, PSM-E-transfected PC3, and LNCaP cells. (**G-H**) Western blotting was performed to detect Flag and exosomal biomarkers (CD63 and Flotillin-2) in purified exosomes derived from 293T and PC3 cells transfected with empty vector and PSM-E-Flag construct. (**I**) Western blotting analysis of the expression of PSM-E in exosome derived from LNCaP cells transfected with PSM-E siRNA or non-targeting control siRNA. CD63 and Flotillin-2 served as the internal control of exosomes. (**J**) The Mass spectrum of a representative peptide fragment of PSM-E protein in exosomes derived from 293T cells

We next sought to investigate whether PSM-E is present in cell-derived exosomes. Specifically, PSM-E was overexpressed in 293T and PC3 cells, which had low basal levels of PSM-E (Supplementary Figure S1A), through transfection with the PSM-E-Flag construct. Subsequently, culture medium supernatants were collected and processed through a series of steps, which included two rounds of centrifugation at 4 °C so as to remove cells and cell debris. The resulting supernatants were further passed through 0.22-µm filters. After this, using a centrifugal filter unit (Amicon Ultra-15) with a 3 kDa cutoff value, the supernatants were concentrated ten-fold. As depicted in Fig. 2A and B, our findings demonstrated the presence of PSM-E-Flag protein both in the cytoplasm and the in culture medium supernatants. Additionally, Western blotting analysis, utilizing a PSM-E-specific antibody, confirmed the presence of PSM-E protein in the supernatant derived from LNCaP cells with high basal levels of endogenous PSM-E (Fig. 2C). Meanwhile, we constructed siRNAs targeting the back-splice region of PSM-E (PSM-E siRNA) to depleted basal-level PSM-E expression in LNCaP cells (Supplementary Figure S1B).

Since a putative signal peptide is not present in the amino acid sequence of PSM-E, we hypothesized that PSM-E is exported via an exosome-mediated mechanism. To validate this hypothesis, we isolated exosomes from the culture media of various cell lines, including 293T cells transfected with PSM-E (293T-PSM-E), 293T cells transfected with control vector (293T-Vector), PSM-E-transfected PC3 cells (PC3-PSM-E), control vector-transfected PC3 cells (PC3-Vector), LNCaP cells, PSM-E siRNA transfected LNCaP cells (LNCaP-siRNA), and a non-targeting control siRNA transfected LNCaP cells (LNCaP-siNC). As shown in Fig. 2D-F, electron microscopy revealed the typical double-layer membrane structure of exosomes with an approximate diameter of 100 nm. Notably, PSM-E was also abundant in those exosomes derived from 293T-PSM-E, PC3-PSM-E and LNCaP-siNC cells. In contrast, exosomes obtained from 293T-Vector, and PC3-Vector did not exhibit detectable levels of PSM-E, and transfection of PSM-E-siRNA efficiently depleted PSM-E expression level in these exosomes (Fig. 2G-I). These results were validated using CD63 and flotillin-2 as exosomal control markers (Fig. 2G-I). The presence of PSM-E in the exosome composition was further confirmed using mass spectrometry (Fig. 2J). Collectively, these data demonstrate that exosome secretion of PSM-E is not limited to LNCaP cells, which express high levels of endogenous PSM-E, but also occurs in 293T and PC3 cells transiently expressing exogenous PSM-E.

### Exosomal PSM-E can be transferred to recipient cells

**Fig. 3:**
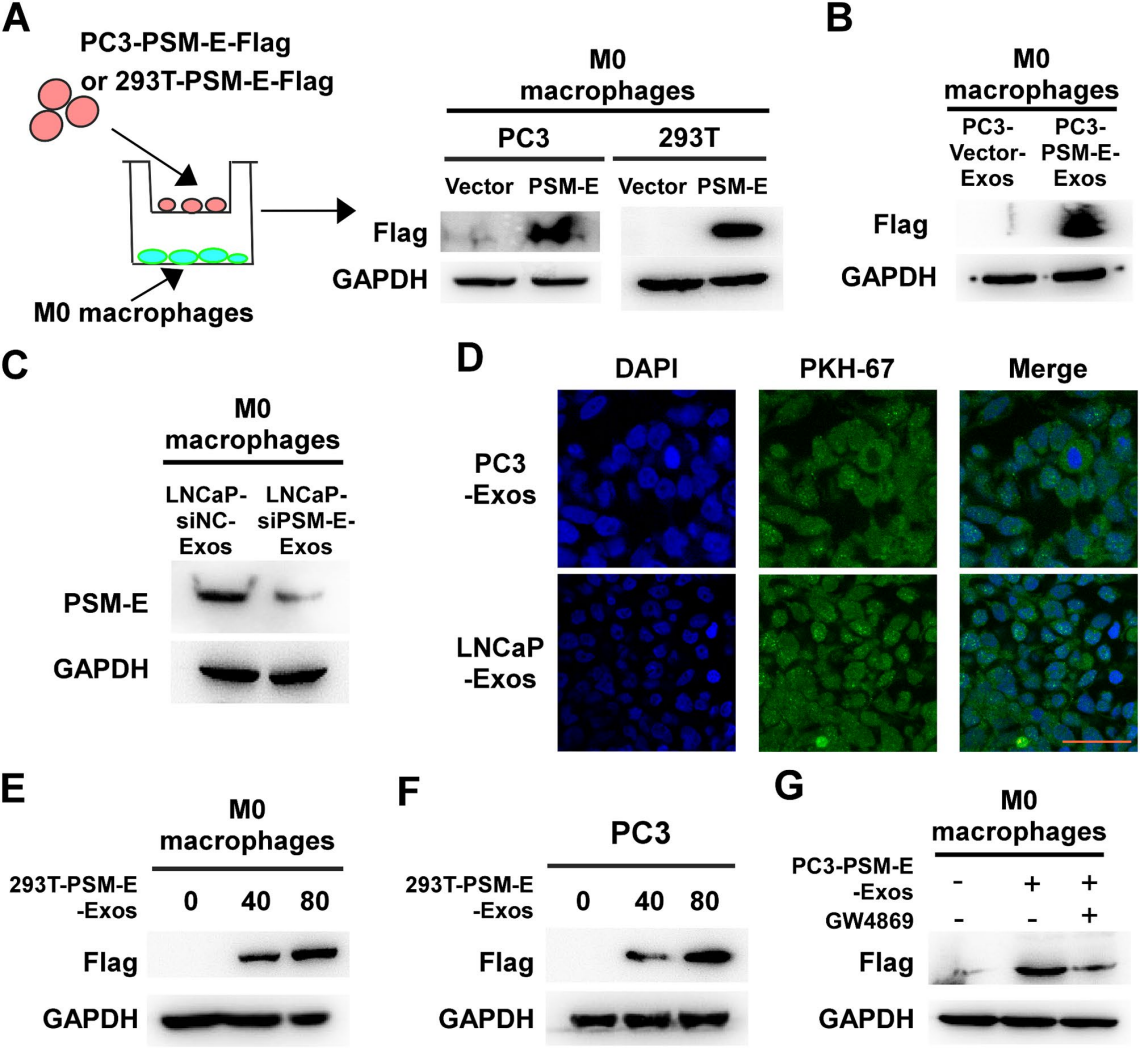
Exosomal PSM-E can be transferred to recipient cells. (**A**) PC3 and 293Tcells transfected with Flag epitope-tagged PSM-E plasmid were co-cultured with M0 macrophages (THP-1 macrophage cells induced by PMA) in a Transwell plate. (**B**, **C**) PSM-E expression in M0 macrophages treated with purified exosomes derived from PC3-Vector, PC3-PSM-E-Flag, LNCaP-siNC, and LNCaP-siPSM-E cells. (**D**) Representative confocal microscopy image of the internalization of fluorescently labeled exosomes in mixed M0 macrophages. Scale bars, 100 μm. (**E**, **F**) PSM-E-Flag expression in M0 macrophages or PC3 cells treated with 0, 40, and 80 µg purified exosomes derived from 293T-PSM-E cells were analyzed by immunoblotting. (**G**) The effects of the EV secretion inhibitor GW4689 (10 µM) on exosome-dependent PSM-E delivery from PC3 cells into M0 macrophages. PSM-E-Flag expression in M0 macrophages treated with purified exosomes derived from PC3-PSM-E cells, followed by treatment with or without GW4689 (10 µM), were analyzed by immunoblotting

Exosomes are protein-containing extracellular vesicles known to play a major role in protein transport among cells. In light of the discovery that PSM-E is contained in exosomes and can be exported from cells expressing it, we investigated whether PSM-E can be transferred to recipient cells. To this end, we tested such a possibility by employed a co-culture system. After transfecting PC3 and 293T cells with a Flag epitope-tagged PSM-E plasmid, these cells were co-cultured with M0 macrophages in the transwell plates (Fig. 3A). Our data demonstrated that the Flag epitope-tagged PSM-E from the upper transwells was delivered into the recipient M0-type THP-1 cells, which were seeded in the lower wells (Fig. 3A), confirming that cells can indeed secrete extracellular PSM-E that is taken up by M0 macrophages.

The remodeling of regional fibroblasts by tumor cells is associated with increased malignant progression and poor outcome in solid tumors such as PCa. Fibroblasts are a key cellular component of human tissues and tumors. To investigate exosomal PSM-E can be transferred to fibroblasts, PC3 and 293T cells were transfected with a Flag epitope-tagged PSM-E plasmid, these cells were co-cultured with HFF-1 in the transwell plates (Figure S2A). Our data demonstrated that the Flag epitope-tagged PSM-E from the upper transwells was delivered into the recipient HFF-1 cells, which were seeded in the lower wells (Figure S2A), confirming that cells can indeed secrete extracellular PSM-E that is taken up by fibroblasts.

Next, we tested whether recipient cells could take up exosomal PSM-E when treated with purified exosomes derived from PSM-E-expressing cells. We differentiated the human monocyte cell line THP-1 into M0-like macrophages using PMA, characterized by their adherent morphology and elevated expression of the macrophage marker CD68 (Supplementary Figure S3A). Subsequently, these M0 macrophages were incubated with exosomes purified from different sources, including PC3-Vector, PC3-PSM-E, LNCaP-siNC, and LNCaP-siPSM-E. As shown in Fig. 3B, an increased abundance of PSM-E in the recipient M0 macrophages treated with exosomes from PC3-PSM-E (PC3-Vector-Exos and PC3-PSM-E-Exos, 80 µg/ml). Also, an increased abundance of PSM-E in the recipient HFF-1 treated with exosomes from PC3-PSM-E (PC3-Vector-Exos and PC3-PSM-E-Exos, 80 µg/ml) (Figure S2B). Additionally, the identification of PSM-E proteins in the lysates of recipient M0 macrophages treated with exosomes from LNCaP-siNC and LNCaP-siPSM-E further confirmed the transfer of PSM-E (Fig. 3C). A fluorescent dye PKH67 was added to the culture medium of M0 macrophages to identify these exosomes derived from cells. After 12 h, the THP-1 cells exhibited efficient uptake of the cell-secreted exosomes, as indicated by the presence of green fluorescence staining in M0 macrophages (Fig. 3D).

Furthermore, to validate that exosomal transfer of PSM-E was responsible for the increase in PSM-E protein levels in recipient cells, PSM-E levels was measured in the recipient cells treated with various concentrations of PSM-E-containing exosomes. As shown in Fig. 3E, F, the finding demonstrated that the levels of PSM-E proteins in the lysates of recipient M0 macrophages and PC3 cells treated with PSM-E-laden exosomes derived from 293T-PSM-E cells (at 40–80 µg) were dose-dependent, as confirmed by immunoblotting. Importantly, when an extracellular vesicle secretion inhibitor, GW4869, was added to the cells 24 h prior, it blocked exosome production and PSM-E delivery into the recipient M0-THP-1 cells, indicating that cells primarily secrete extracellular PSM-E in an exosome-dependent manner (Fig. 3G). Overall, these results demonstrate that prostate cancer cells can secrete PSM-E-containing exosomes efficiently taken up by recipient cells.

### Exosomal PSM-E inhibits PCa cell migration, invasion, and the polarization of M0 macrophage to the M2 phenotype

**Fig. 4:**
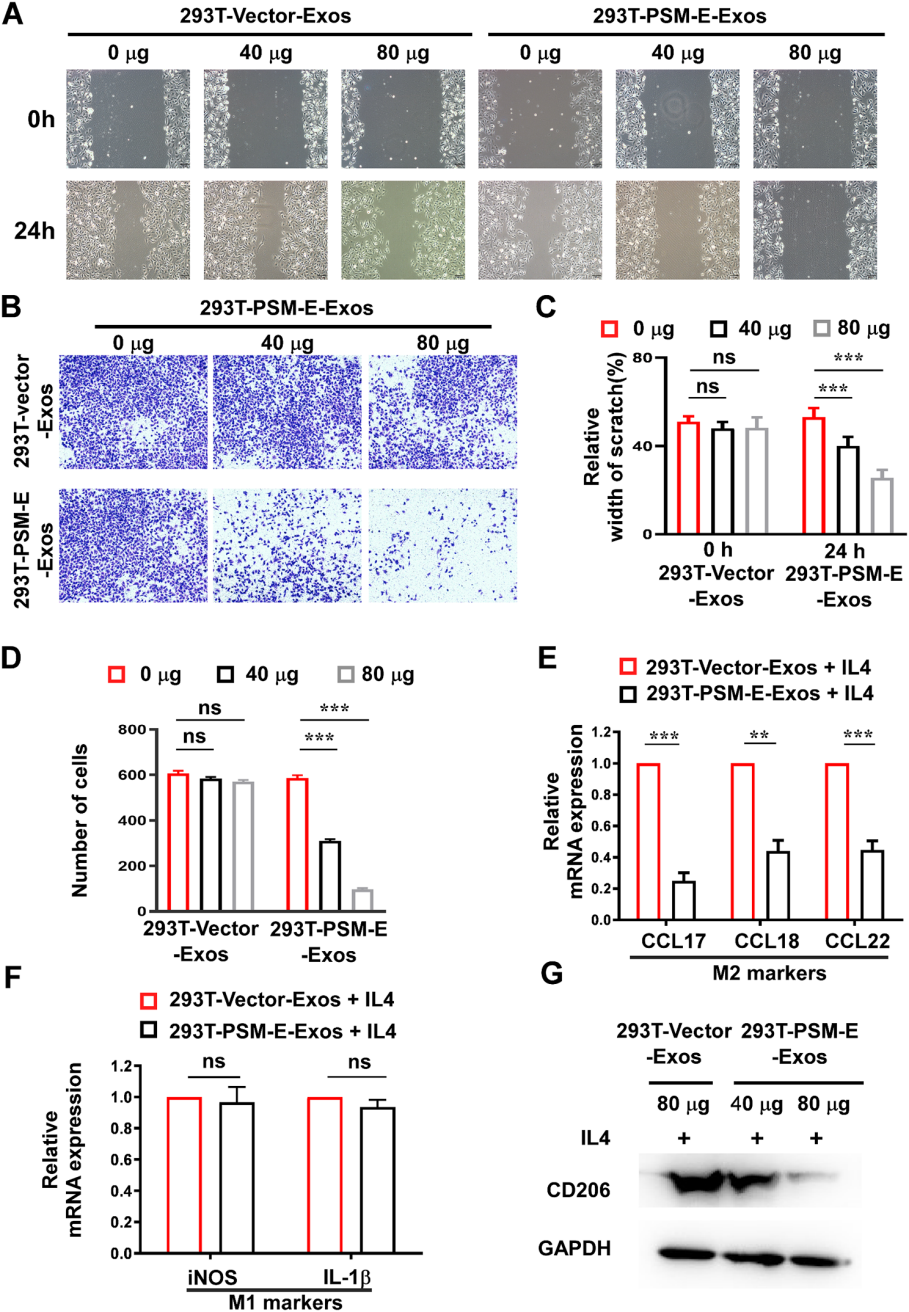
Exosomal PSM-E inhibits PCa cell invasion and M2 polarization of macrophage. (**A**, **B**) Cell migratory and invasive ability of PC3 cells upon treatment with 40, 80 µg of 293T-PSM-E-Exosomes analyzed by wound healing assay and Matrigel-coated transwell assays. Scale bar, 200 μm. (**C**) Cell migration quantified as a percentage of the wound-healed area. (**D**) Average number of invading cells per field from three independent experiments. (**E**, **F**) M0 macrophages were incubated with IL4 (20 ng/ml), in combination with purified exosomes derived from 293T-PSM-E cells or 293T-Vector cells for 48 h. Cells were collected, and the mRNA expression of M2 markers, including CCL17, CCL18, and CCL22 mRNA, were qualified by real-time RT-PCR. Meanwhile the mRNA expression of M1 markers including iNOS and IL-1β was detected by real-time RT-PCR (*p < 0.05, **p < 0.01 and ***p < 0.001; compared with the control group). (**G**) M0 macrophages were incubated with IL4 (20 ng/ml) in combination with 293T-PSM-E-Exos (40, 80 µg) or 293T-vector-Exos (80 µg) for 48 h. Cells were collected and subjected to Western blotting analysis to detect the protein levels of M1 marker CD206

Our previous studies revealed that PSM-E could suppress the proliferation, migration, and invasiveness of prostate cancer cells [23, 24]. To explore the effects of exosomal PSM-E on tumor migration and invasion, we conducted wound healing assays and transwell invasion experiments on recipient PC3 cells treated with exosomes derived from 293T-PSM-E and 293T-Vector cells at different concentrations (40–80 µg/ml). The results indicated that 293T-PSM-E-Exosomes inhibited PC3 cell migration and invasion compared to 293T-Vector-Exosomes (Fig. 4A-D). These results suggest that exosomal PSM-E can also suppress the invasive and metastatic abilities of PC3 cells.

Our previous findings revealed that PSM-E was specifically overexpressed in PCa and its expression is negatively correlated with the expression of CD206 (a specific marker of M2-type macrophages), which suppressing the secretion of inflammatory cytokines in the PCa microenvironment and inhibiting the chemotaxis of monocytes [28]. In this study, we focused on investigating PSM-E’s potential impact on the tumor microenvironment in the context of PCa progression [28]. Additionally, M0 macrophages were treated with IL-4 to induce their differentiation into M2-like macrophages (Supplementary Figure S3B). Real-time RT-PCR analysis revealed a significant increase in the levels of M2 markers, including CCL17, CCL18, and CCL22, in M2 macrophages (Supplementary Figure S3C). Subsequently, we examined the effects of 293T cell-derived exosomal PSM-E on the polarization of M0 macrophages towards the M2 phenotype. The expression levels of M2 markers (CCL17, CCL18, and CCL22) markedly decreased in M0 macrophages co-treated with 293T-PSM-E-Exos and IL-4, compared to those in the 293T-vector-Exos and IL-4 treatment groups (Fig. 4E), suggesting that the proportion of M2-type macrophages in THP-1 cells was reduced following treatment with PSM-E exosomes. In contrast, there was no change in the expression of M1 macrophage markers (iNOS and IL-1β) after similar treatment with 293T cell-derived exosomal PSM-E (Fig. 4F). As shown in Fig. 4G, our results demonstrated that the levels of CD206 proteins in the lysates of recipient THP-1 macrophages (THP-1 treated with PMA and IL-4) exposed to PSM-E-laden exosomes derived from 293T-PSM-E-cells (40 µg, 80 µg) exhibited a dose-dependent decrease, as determined by immunoblotting. In summary, the above findings confirm that cell-derived exosomal PSM-E can suppress the M2 polarization of macrophages.

### PSM-E colocalizes and interacts with RACK1

**Fig. 5:**
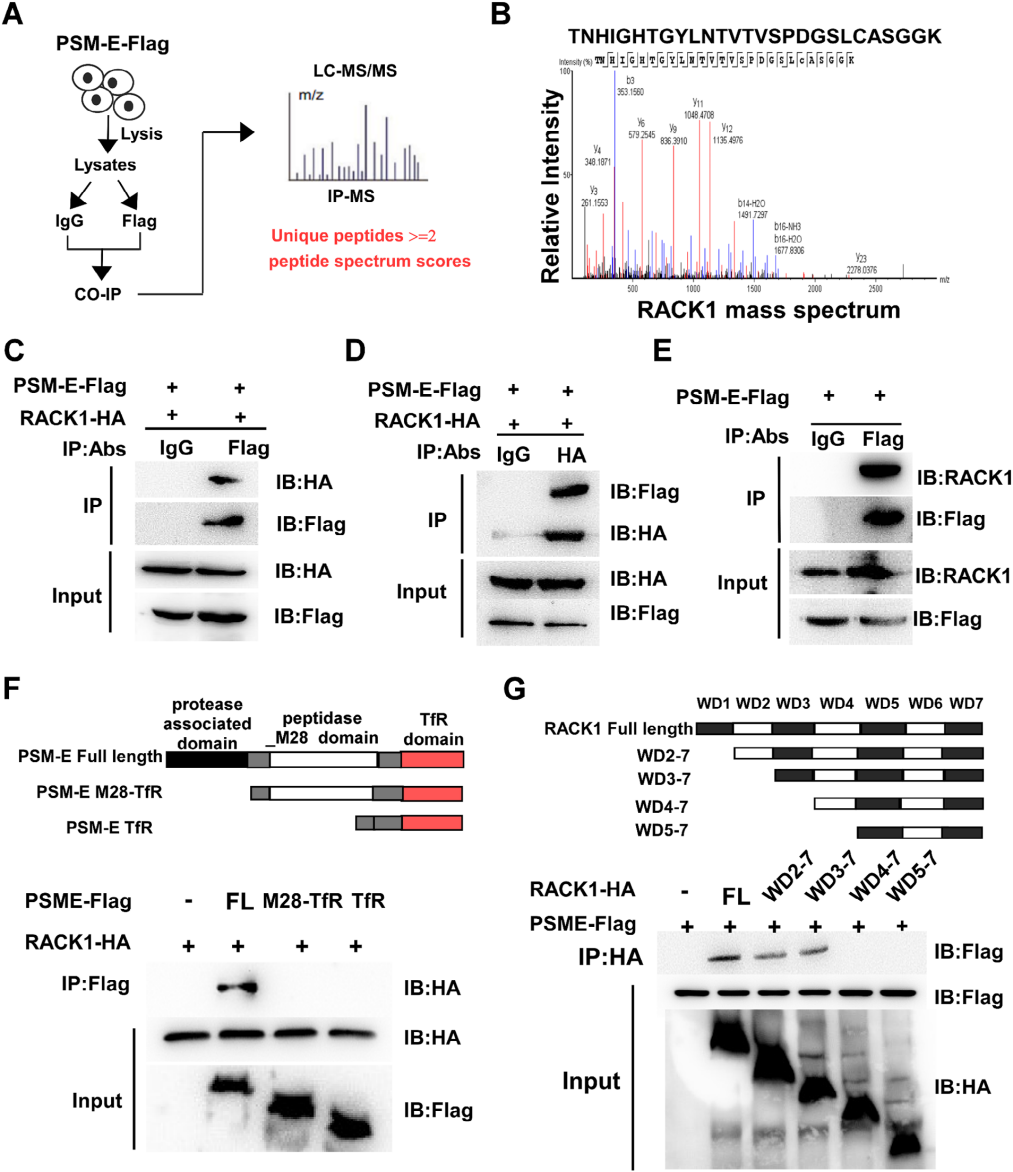
PSM-E colocalizes and interacts with RACK1. (**A**) Flow diagram showing immunoprecipitation (IP) of Flag-PSM-E protein and subsequent LC-MS/MS analysis. (**B**) The mass spectrum of a representative peptide fragment of PSM-E protein. (**C**, **D**) 293T cells co-transfected with plasmids encoding Flag-tagged PSM-E and HA-tagged RACK1 were used for a co-IP assay. Cell lysates were precipitated with an anti-Flag antibody (**C**), anti-HA antibody (**D**) or control IgG, and immunocomplexes were analyzed with the indicated antibodies by Western blotting. (**E**) PC3 cells were transfected with a plasmid encoding Flag-tagged PSM-E, followed by immunoprecipitation using an anti-Flag antibody or control IgG. The immunocomplexes were analyzed with anti-RACK1 antibody by Western blotting. (**F**) A schematic diagram of the PSM-E protein and functional domains: protease associated domain (PA) and peptidase M28 domain (M28). Co-IP and western blotting analysis of 293T cells transfected with HA-tagged RACK1 along with vectors expressing the indicated Flag-tagged PSM-E truncation forms or full-length PSM-E. (**G**) A schematic diagram of the RACK1 protein and functional domains. Five serial WD domain deletion constructs were generated, resulting in full-length (FL) plasmids, WD2-7, WD3-7, WD4-7, WD5-7. Co-IP and western blotting analysis of 293T cells transfected with Flag-tagged PSM-E along with vectors expressing the indicated HA-tagged RACK1 truncation forms or full-length HA

We further sought to explore the mechanisms underlying the inhibitory effect of PCa-derived exosomal PSM-E on the M2 polarization of macrophages. To this end, using the protein G-conjugated IgG antibody or anti-Flag monoclonal antibody, lysates from PC3-PSM-E-Flag cells were immunoprecipitated (Fig. 5A), followed by a pull-down experiment, and subsequently analyzed the immunoprecipitates by LC-MS/MS (Fig. 5A). We screened the putative interacting proteins with PSM-E obtained from IP-MS analysis based on the unique peptides (unique peptides ≥ 2) and peptide spectrum scores ranking (Fig. 5A). Notably, RACK1 was picked out as a potential candidate protein, and the mass spectrometer of the RACK1 peptide segment was depicted in Fig. 5B. To validate that PSM-E interacts with RACK1, we performed co-immunoprecipitation (co-IP) assays after co-transfection of PSM-E-Flag and RACK1-HA expression plasmids in 293T cells. Significantly, as shown in Fig. 5C and D, our results showed that RACK1-HA was precipitated by the Flag antibody, meanwhile, PSM-E-Flag was precipitated by the HA antibody, revealing the formation of a complex between PSM-E-Flag and RACK1-HA. Furthermore, the interaction between Flag-tagged PSM-E and RACK1 was investigated using an IP assay using endogenous RACK1 as a precipitate, which resulted in the successful pull-down of endogenous RACK1 together with Flag-tagged PSM-E (Fig. 5E). Moreover, to assess whether endogenous PSM-E interacts with RACK1, LNCaP cells co-transfected with siRNAs targeting PSM-E and plasmid encoding and HA-tagged RACK1 were used for co-IP assay. As shown in Supplementary Figure S4, our results showed that almost none of the PSM-E protein was precipitated by the HA antibody.

To identify the specific domains of PSM-E responsible for its interaction with RACK1, we generated two deletion mutants that removed the protease-associated domain (PA) and the peptidase M28 domain (M28) of PSM-E. Co-IP assays revealed that the protease-associated domain of PSM-E was crucial for its interaction with RACK1 (Fig. 5F). It is well-established that RACK1 belongs to the tryptophan- aspartate repeat (WD-repeat) proteins family which share significant homology with the β subunit of G-proteins. We generated five serial WD domain deletion constructs, including full-length (FL), WD2-7, WD3-7, WD4-7, and WD5-7 (Fig. 5G). Co-IP assays using an anti-HA antibody and subsequent immunoblotting with an anti-Flag antibody showed that the fourth WD domain of RACK1 was essential for its interaction with PSM-E (Fig. 5G). Taken together, these results indicate that the protease-associated domain of PSM-E and the fourth WD domain of RACK1 are crucial for the interaction between PSM-E and RACK1.

### Exosomal PSM-E inhibits M2 polarization of macrophages via suppression of the ERK and FAK signaling pathways

**Fig. 6:**
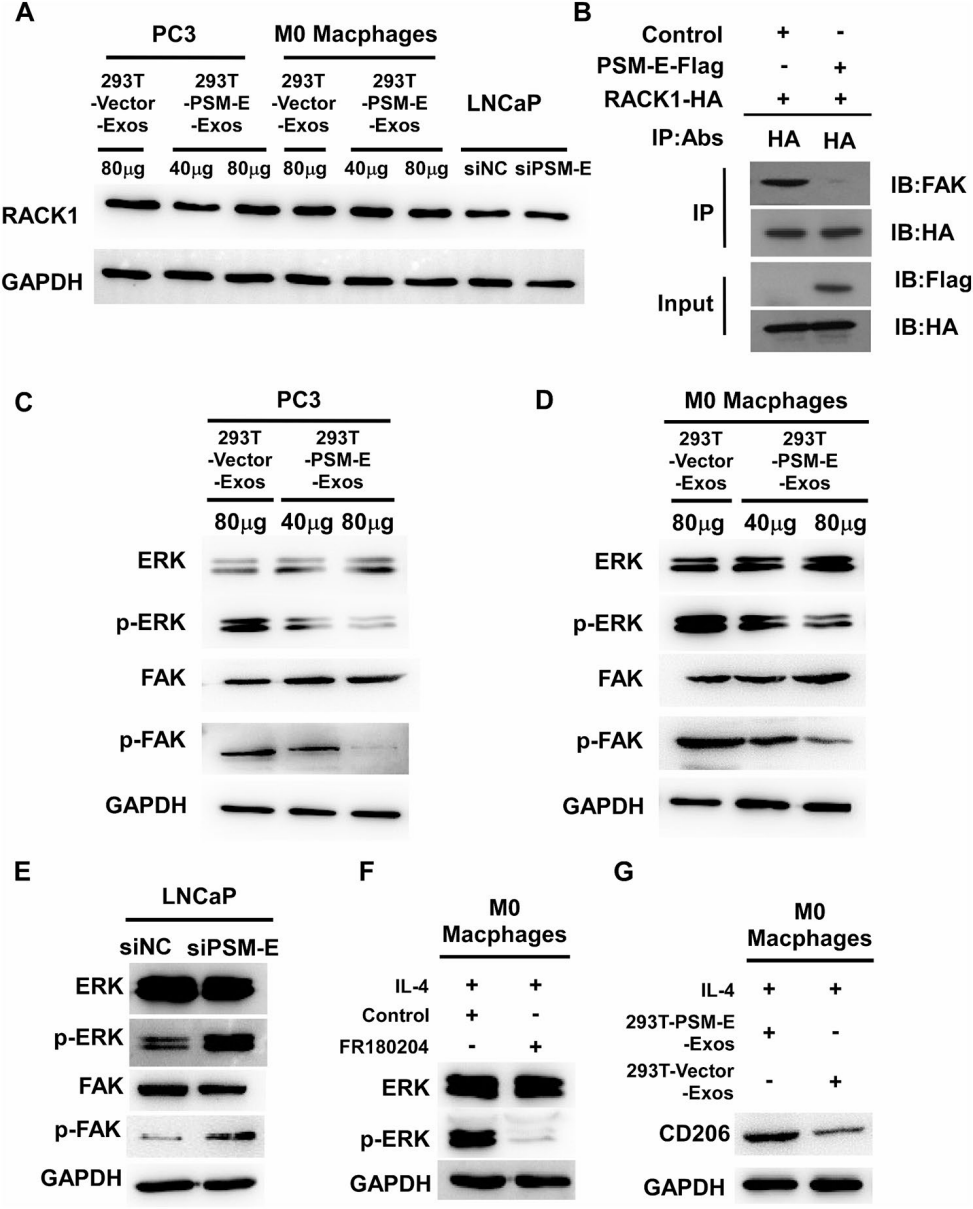
Exosomal PSM-E inhibits M2 polarization of macrophages by suppressing the RACK1-ERK/FAK signaling pathway. (**A**) Western blotting analysis of PCa cells (PC3 and LNCaP cells) and macrophages treated with 293T-PSM-E-Exos or siPSM-E. (**B**) 293T cells were transfected with HA-tagged RACK together with or without Flag-tagged PSM-E. The cell lysates were immunoprecipitated with anti-HA antibody and analyzed by Western blotting with anti-FAK antibody, anti-HA or anti-Flag antibody. (**C**, **D**) PC3 cells or M0 macrophages were treated with different concentrations of purified exosomes derived from 293T-PSM-E cells for 48 h and then harvested for immunoblotting analysis. The phosphorylation levels and total expression of the ERK and FAK proteins were verified by western blotting analysis. GAPDH was used as an internal control. (**E**) Effect of PSM-E on the ERK and FAK signaling pathway in LNCaP cells with PSM-E knockdown. Cells were transfected with PSM-E-siRNA or Scrambled siRNA for 48 h. Western blotting analysis was performed using antibodies against phospho-ERK and FAK, with GAPDH used as a loading control. (**F**) M2 macrophages were incubated with or without FR180204 (10µM) for 48 h, and then, the phosphorylation levels and total expression of the ERK proteins were verified by western blotting analysis. GAPDH was used as an internal control. (**G**) M2 macrophages were treated with 80 µg of purified exosomes derived from 293T-Vector and 293T-PSM-E cells for 48 h and then harvested for immunoblotting analysis. CD206 protein was verified by western blotting assay was carried out. GAPDH was used as an internal control

Numerous studies have indicated that the activation of the ERK signaling pathway in macrophages is involved in the M2 polarization of macrophages [32]. Moreover, RACK1 has been reported to regulate the ERK and FAK pathway in various cellular activities [33]. To gain a deeper understanding of the RACK1 signaling cascades that mediate the inhibitory effect of exosomal PSM-E on M2 macrophage polarization, we examined changes in the phosphorylation status of RACK1 downstream substrates, such as ERK and FAK, using Western blotting. Our current data identified that PSM-E interacted with RACK1, thereby prompting us to investigate whether PSM-E can alter the expression levels of RACK1. As shown in Fig. 6A, Western blotting analysis of PCa cells (PC3 and LNCaP cells) and macrophages treated with 293T-PSM-E-Exos or siPSM-E, showed that PSM-E did not affect the expression level of RACK1 protein. To investigate whether PSM-E interfere with the binding of RACK1 to its substrate FAK, we examined the formation of the RACK1-FAK complex in the presence or absence of PSM-E respectively. 293T cells were transfected with HA-tagged RACK together with or without Flag-tagged PSM-E. The cell lysates were immunoprecipitated with anti-HA antibody and analyzed by Western blotting with anti-FAK antibody or anti-HA antibody. We found that RACK strongly interacted with FAK in the absence of PSM-E, whereas cells expressing PSM-E exhibited a significant decrease in the interaction between RACK1 and FAK protein (Fig. 6B). As shown in Fig. 6C and D, exosomal PSM-E led to a significant decrease in the levels of phosphorylated FAK and ERK (p-FAK and p-ERK) without affecting the expression of total ERK and FAK in both PC3 and M0 macrophages (THP-1 macrophage cells induced by PMA) in a dose-dependent manner. In contrast, the attenuation of PSM-E expression in LNCaP cells through transfection with PSM-E siRNA (siPSM-E) reversed the activation of the ERK/FAK pathway (Fig. 6E). Notably, M2-like macrophages treated with FR180204 (an ERK inhibitor) for 12 h exhibited a significant decrease in phosphorylated ERK levels without affecting the expression of total ERK (Fig. 6F). As expected, CD206 expression decreased in M2 macrophages treated with 293T-PSM-E-Exos (Fig. 6G). These results suggest that exosomal PSM-E could inhibit M2 macrophage polarization via suppressing the RACK1- FAK-ERK signaling pathway.

### Exosomal PSM-E PCa growth and correlates with low infiltration of M2 macrophage in vivo

**Fig. 7:**
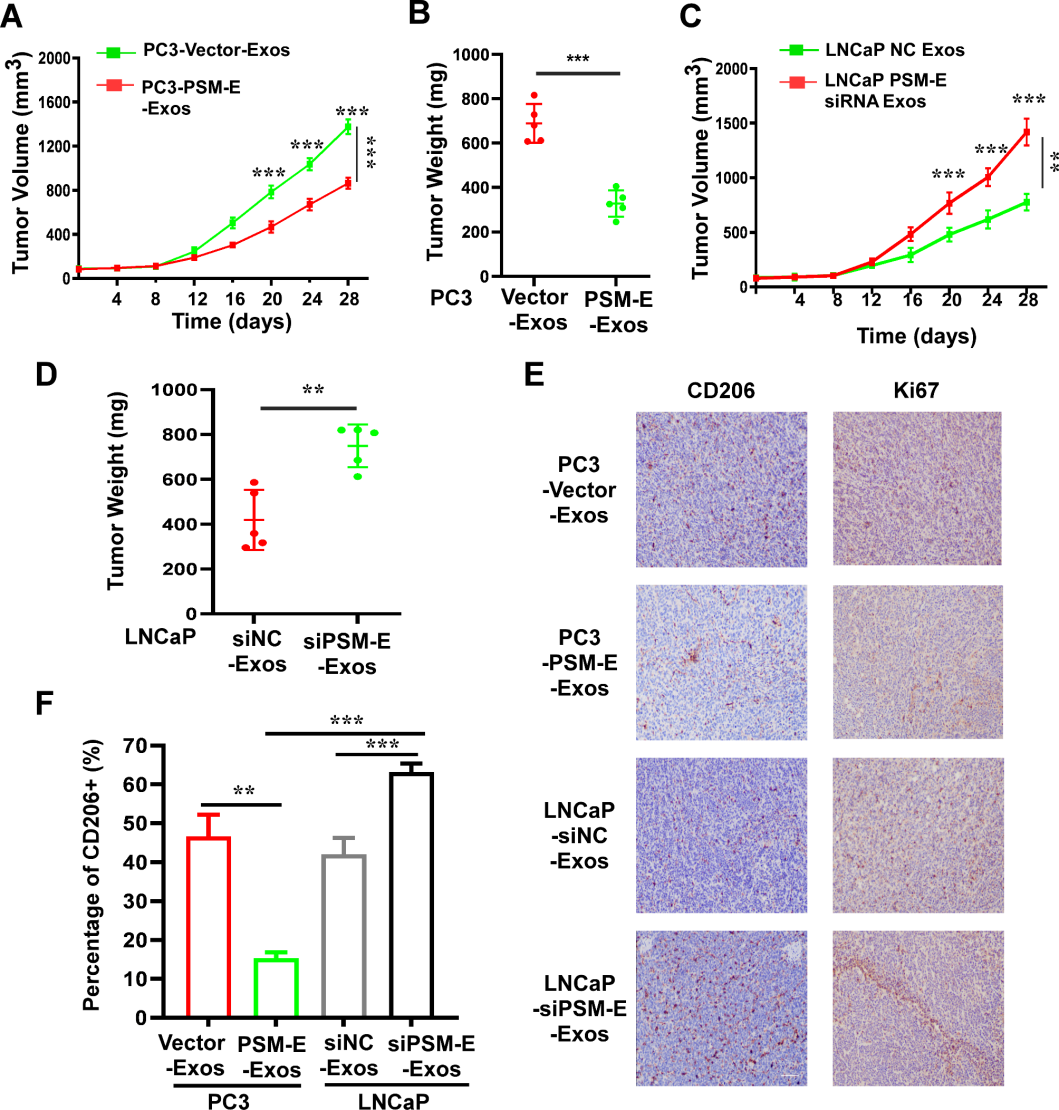
Exosomal PSM-E restrains tumor growth of PCa and correlates with low M2 macrophage abundance in vivo. (**A**) Time-response curve of the effect of purified exosomes derived from PC3-Vector and PC3-PSM-E cells on the growth of homograft RM-1 tumor. (**B**) Tumor weights were measured after the tumors were surgically dissected (*n* = 5) in PC3-Vector and PC3-PSM-E-Exos groups. (**C**) Time-response curve of the effect of purified exosomes derived from LNCaP-siNC and LNCaP-siPSM-E cells on the growth of homograft RM-1 tumor. (**D**) Tumor weights were measured after the tumors were surgically dissected (*n* = 5) in LNCaP siNC-Exos and siPSM-E-Exos groups. (**E**) IHC examination of CD206 and Ki67 expression in the tumor tissues for each group as indicated. Scale bars: 100 μm. (**F**) Statistical analysis of the percentage of CD206^**+**^ in the mouse tumor tissues. Results are presented as means ± SDs are provided. The asterisks indicate statistically significant differences as compared with the control, with **, and *** indicating *p* < 0.05, *p* < 0.01, and *p* < 0.001

To assess the anti-PCa activity of exosomal PSM-E in vivo, we established a subcutaneous PCa tumorigenesis model in immunocompetent C57BL/6J mice. In this model, we subcutaneously injected RM-1 cells into C57BL/6J mice to develop solid tumors until the tumors reached a volume of approximately 50 mm^3^ (Supplementary Figure S5A). We then monitored the tumor growth after intraperitoneal injections of exosomes derived from PC3 cells with exogenously overexpressed PSM-E (PC3-Vector-Exos, PC3-PSM-E-Exos) or LNCaP cells with high basal levels of endogenous PSM-E (LNCaP-siNC-Exos and LNCaP-siPSM-E -Exos) every three days for four weeks (Supplementary Figure S5A). As shown in Supplementary Fig. 7A and Figure S5B, the volume of the homograft PCa tumors was significantly reduced by 43.6% in mice treated with PC3-PSM-E-Exos. The weight of the homograft tumors at the experimental endpoint was also notably reduced by 49.12 ± 3.81% in C57BL/6 mice treated with PC3-PSM-E-Exos (Fig. 7B). Conversely, compared to the LNCaP-siNC-Exos group, the volume and weight of tumors derived from the LNCaP-siPSM-E-Exos group were significantly increased (Fig. 7C, D and Figure S5C). IHC analysis of mouse homograft tumors revealed a significantly reduced expression of CD206^+^ (a specific marker of M2-type tumor-associated macrophages) in the PC3-PSM-E-Exos treatment group, while a higher expression of CD206^+^ was observed in the LNCaP-siPSM-E-Exos treatment group (Fig. 7E). Furthermore, the results revealed higher expression of the proliferation marker Ki67 in the tumors derived from the LNCaP-siPSM-E-Exos group compared to those in the PC3-PSM-E-Exos group (Fig. 7E). These findings suggest that exosomal PSM-E can suppress tumor growth and correlate with a lower infiltration of M2 macrophages in vivo.

## Discussion

It is now understood that metastasis is the leading cause of PCa-related mortality and results from complex interactions between the tumor microenvironment and tumor cells within the prostate [34], which drives tumor progression and distant spread. Herein, we demonstrated the presence of PSM-E-enriched exosomes in the urine of PCa patients, which significantly correlated with PCa tumor grade. Importantly, we uncovered that PSM-E-enriched exosomes could hinder PCa progression by facilitating communication between PCa cells and TAMs in the metastatic microenvironment. We also elucidated the underlying mechanism of PSM-E-triggered TAM polarization, revealing that RACK1, an integral component of PSM-E, suppresses the ERK and FAK signaling pathways during M2 polarization. The involvement of exosomal PSM-E in the interplay between TAMs and tumor cells provides insights into the molecular mechanisms of PCa proliferation and metastasis and suggests that it may serve as a novel marker for liquid biopsy in PCa.

It is well-established that a large proportion of PCa cases diagnosed through PSA screening are not clinically significant due to a high false-positive rate. Consequently, there is an urgent need for development of novel biomarkers to enhance the accuracy of PCa diagnosis. Current evidence suggests that exosomes can transfer proteins, RNAs, DNA, lipids, and metabolites, making them promising surrogates for tissue biopsy [35–37]. Thus, exosome isolated from body fluids, such as serum, saliva, and urine, from cancer patients, have emerged as important alternative biomarkers for tumor early diagnosis and prognosis prediction, as they carry host oncogenes and tumor suppressor genes [38, 39]. Recently, the exosomes in blood, saliva or urine from patients suffering from different cancers have been frequently reported to be liquid biopsy sources for cancer detection [40, 41, 42]. It is reported that that tumor cell exosomes could regulate monocyte M2 polarization through integration of αvβ6 integrin by cycling intracellularly in PCa progression [18]. Nonetheless, their finding solely showed that the αvβ6 integrin has profound effects on the microenvironment by preventing induction of the STAT1/MX1/2 signaling pathway in donor cancer cells and their exosomes, and the investigation of exosomal αvβ6 integrin in PCa patients was not conducted. Future research should consider integrating the detection of exosomal PSM-E and αvβ6 integrin to enhance the diagnostic accuracy of PCa. Additionally, further investigations could examine the potential of the PSM-E to αvβ6 integrin ratio in urine-derived exsomes as a valuable biomarker for the clinical diagnosis and prognosis of PCa.Our current study demonstrated for the first time that the presence of PSM-E-containing exosomes in the extracellular environment, including serum and urine from patients with PCa. We found that the PCa group had significantly higher urine-derived exosomal PSM-E concentrations than the control group, also with markedly higher levels of exosomal PSM-E compared to those with BPH. Furthermore, a positive correlation was found between high levels of exosomal PSM-E and high Gleason scores, advanced pathological tumor stages. Notably, urine-derived exosomal PSM-E yielded an AUC of 0.8904 in diagnosing PCa. These findings suggest that urine-derived exosomal PSM-E represents a novel, non-invasive indicator for early PCa diagnosis and progression prediction, contributing to the development of non-invasive liquid biopsy techniques in the future. Consequently, it is of great interest to investigate whether increased urine-derived exsomal PSM-E levels in human subjects may predispose to preventing PCa progression in larger cohorts. Therefore, further research with a larger sample size in prostatitis, benign prostatic hyperplasia (BPH), and prostate cancer patients etc. are being conducted in our laboratory.

Emerging evidence has highlighted the role of TAM recruitment and M2 polarization in tumor growth, invasion, and metastasis [43]. Macrophages in the tumor microenvironment typically display an M2-like phenotype, promoting the growth, metastasis, and invasion of prostate cancer cells [43]. Notably, tumor-derived exosomes, as a primary source of environmental signals, have been shown to induce macrophages to adopt an M2-like phenotype [43, 44]. Thus, the cargo within exosomes regulates highly efficient communication between tumor cells and macrophages. Our study demonstrated the PSM-E-containing exosomes was found in the tumour extracellular environment, and unveiled their capacity to transport PSM-E between cells, thereby conveying its anti-tumor impact. Our findings showed that PSM-E-containing exosomes could suppress PCa cell invasion and metastasis in vitro. Intriguingly, in a prostate cancer mouse model, exosomal PSM-E significantly inhibited tumor growth. Furthermore, our study demonstrated that high PSM-E expression and M2-type TAMs exhibited opposing trends in both cellular and animal models, consistent with our previous findings in clinical PCa tissues [28]. Based on these findings, it is highly conceivable that PSM-E-containing exosomes inhibit M2 macrophage polarization, attenuating the pro-tumor effect and suggesting PSM-E as a promising therapeutic target for PCa progression.

At the molecular level, we identified RACK1 as the binding partner of PSM-E. It has been established that PSM-E contains a protease-associated domain (150–248), peptidase-28 domain (355–546), and TfR domain (639–703). We thus deleted the protease-associated domain and peptidase-28 domain of PSM-E and found that the protease associated domain of PSM-E is indispensable for its interaction with RACK1. Meanwhile, RACK1 is a 36-kDa protein with seven tryptophan-aspartate repeat (WD-repeat) domains that mediate protein-protein interactions [33]. We further examined which portions of RACK1 were critical for binding to PSM-E, and the co-IP experiments indicated that the fourth WD domain was crucial for their interaction. This domain is also required for membrane localization, indicating that PSM-E may exert its function by interacting with and influencing RACK1. RACK1 is a highly conserved WD40 repeat scaffold protein known to bind to many proteins, thereby impacting multiple signal transduction pathways through compartmentalization [33]. RACK1 is able to integrate inputs from different signaling pathways because WD40 repeats interact with different signaling molecules simultaneously, including ERK signaling [45] and FAK signaling [46].

## Conclusions

**Fig. 8:**
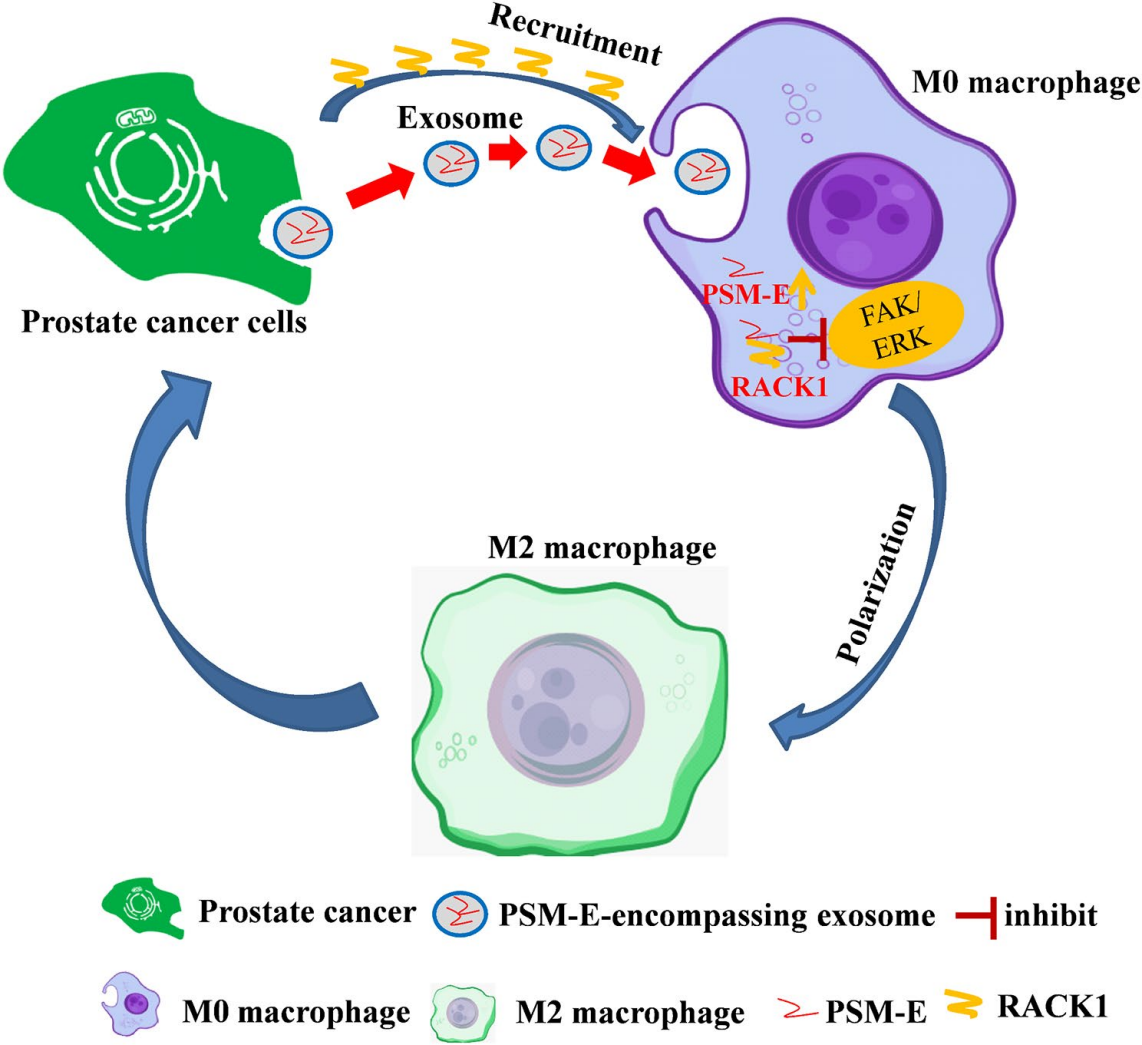
Schematic image of the prostate cancer cell-derived exosomal PSM-E suppressed tumor metastasis by modulating the RACK1/FAK/ERK signaling pathway

Our findings provided evidence of how PCa cell-derived exosomal PSM-E suppressed tumor metastasis by modulating the RACK1/FAK/ERK signaling pathway (Fig. 8). We also revealed that serum and urine exosomal PSM-E could serve as a marker for metastatic disease and prognostic prediction in PCa patients. Our study uncovered the crucial mechanism of exosomal PSM-E-mediated intercellular communication from PCa cells to the tumor microenvironment, endowing common PCa cells with metastatic features, paving the way for a potential non-invasive diagnostic approach for PCa. Notably, targeting exosomal PSM-E may represent an innovative therapeutic strategy for preventing PCa progression.

## Electronic supplementary material

Below is the link to the electronic supplementary material.

Supplementary Material 1

Supplementary Material 2

Supplementary Material 3

Supplementary Material 4

Supplementary Material 5

Supplementary Material 6

## Data Availability

No datasets were generated or analysed during the current study.
