# Correction: Exosomal PSM-E inhibits macrophage M2 polarization to suppress prostate cancer metastasis through the RACK1 signaling axis

**DOI:** 10.1186/s40364-024-00708-4

**Published:** 2024-12-18

**Authors:** Xingliang Qin, Ruoxi Niu, Yongyao Tan, Yuxin Huang, Weishu Ren, Weiwei Zhou, Huiquan Wu, Junlong Zhang, Mingze Xu, Xiang Zhou, Hongyu Guan, Xun Zhu, Yu Chen, Kaiyuan Cao

**Affiliations:** 1 Department of Endocrinology, The First Affiliated Hospital of Sun Yat-sen University, Guangzhou, 510080 China; 2 School of Biomedical Sciences, The Chinese University of Hong Kong, Hong Kong, 999077 China; 3 Key Laboratory of Tropical Disease Control (Sun Yat-sen University), Ministry of Education, Guangzhou, 510080 China; 4 Research Center for Clinical Laboratory Standard, Department of Immunology and Microbiology, Zhongshan School of Medicine, Sun Yat-sen University, 74 Zhongshan Road II, Guangzhou, 510080 China; 5 Department of Urology, The First Affiliated Hospital of Sun Yat-sen University, Guangzhou, 510080 China; 6 Guangzhou Jishiyuan Bio-technology Co., Ltd, Guangzhou, 510700 China; 7 Department of Microsurgery, Trauma and Hand Surgery, The First Affiliated Hospital, Sun Yat-sen University, Guangzhou, 510080 China

**Biomarker Research (2024) 12:138**

10.1186/s40364-024-00685-8

The original article initially erroneously presented co-author, Ruoxi Niu’s name as ‘Rouxi Niu’; this has since been amended.

