# Supplementary figures and images for: Exosomal PSM-E inhibits macrophage M2 polarization to suppress prostate cancer metastasis through the RACK1 signaling axis
Source: Biomark Res. 2024 Nov 14;12:138. doi: 10.1186/s40364-024-00685-8 (PMC11562865; doi:10.1186/s40364-024-00685-8)

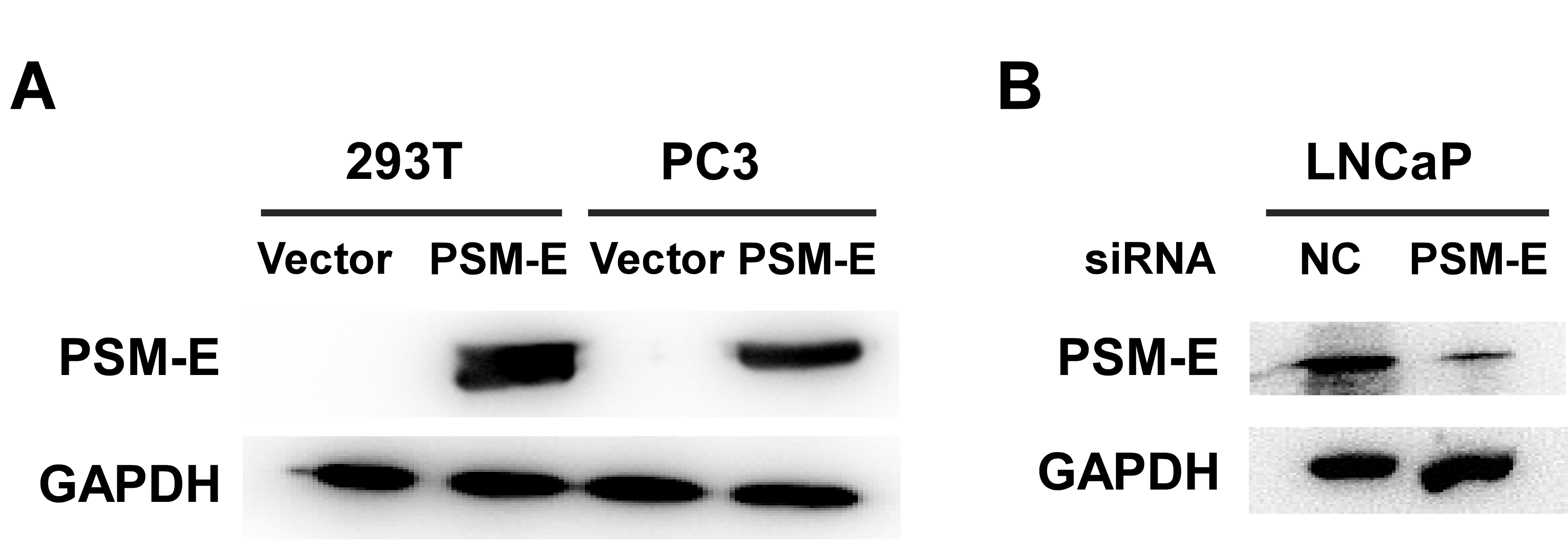

Supplement: Supplementary file 1 — Supplementary Material 1 [file 40364_2024_685_MOESM1_ESM.tif]

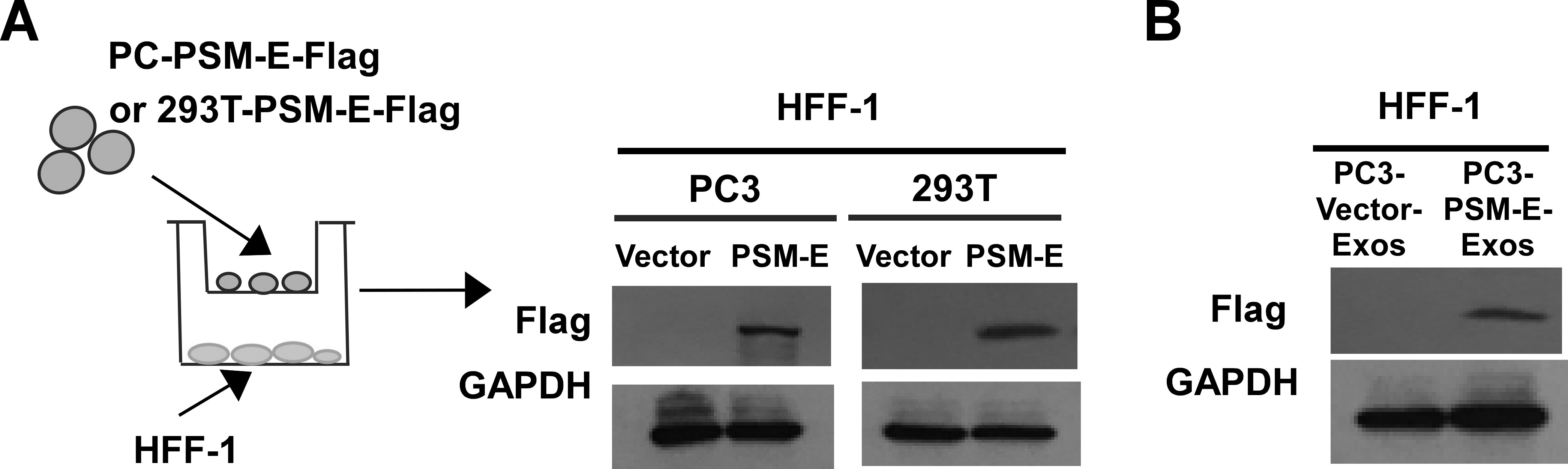

Supplement: Supplementary file 2 — Supplementary Material 2 [file 40364_2024_685_MOESM2_ESM.tif]

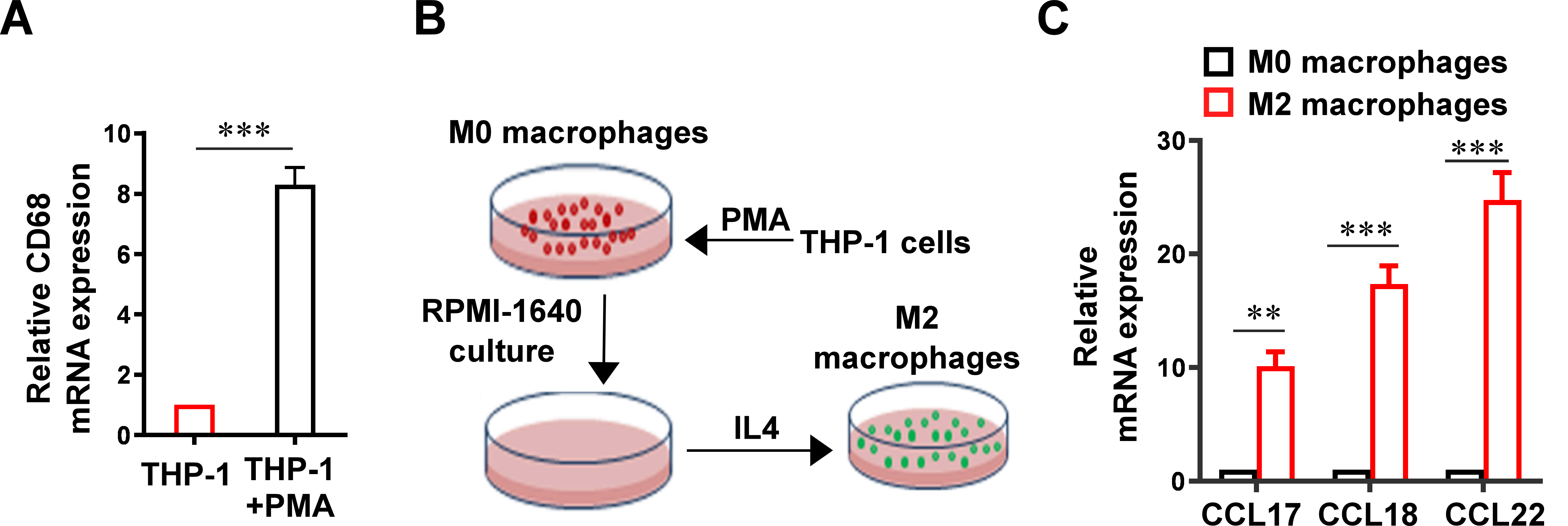

Supplement: Supplementary file 3 — Supplementary Material 3 [file 40364_2024_685_MOESM3_ESM.tif]

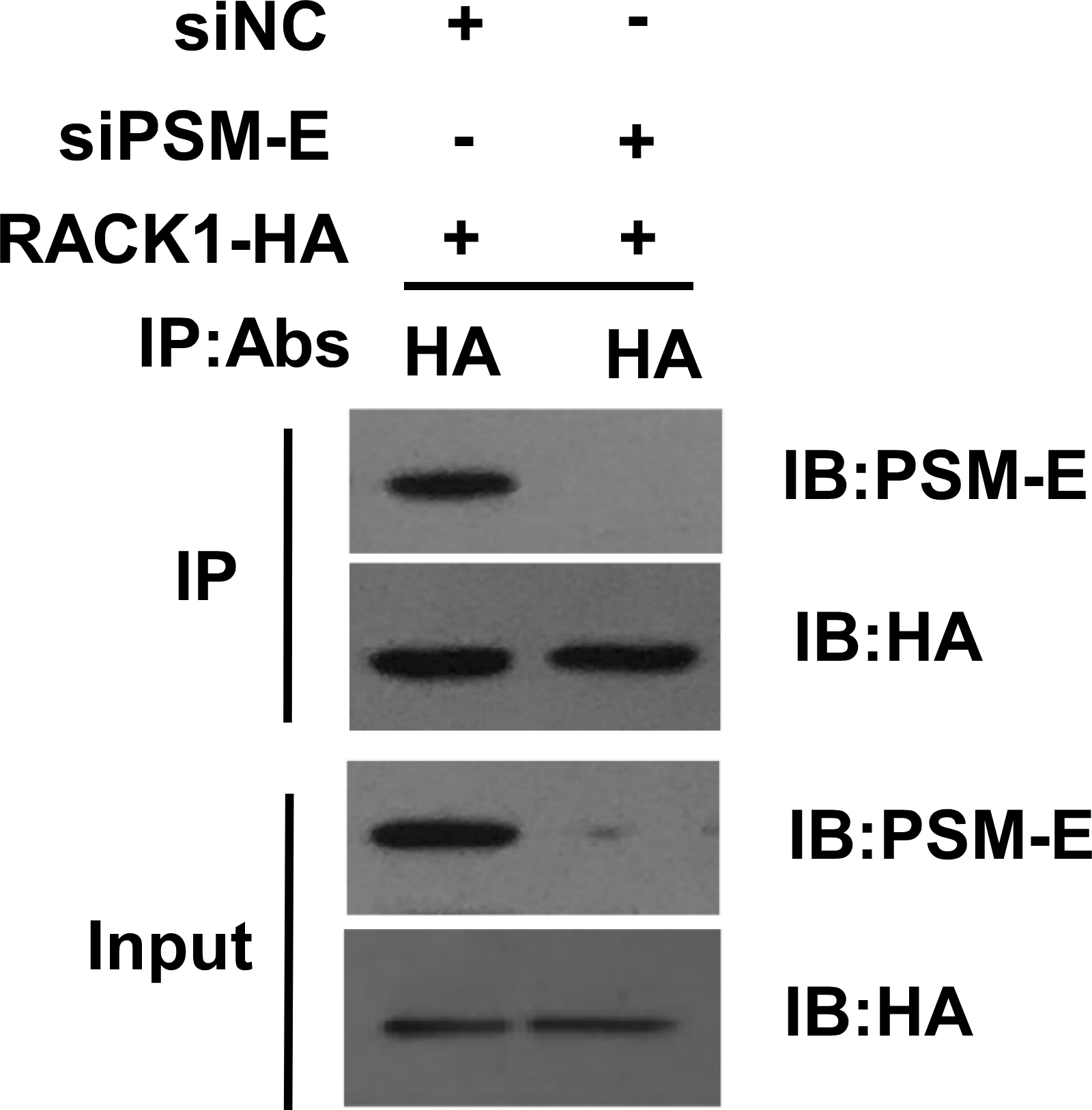

Supplement: Supplementary file 4 — Supplementary Material 4 [file 40364_2024_685_MOESM4_ESM.tif]

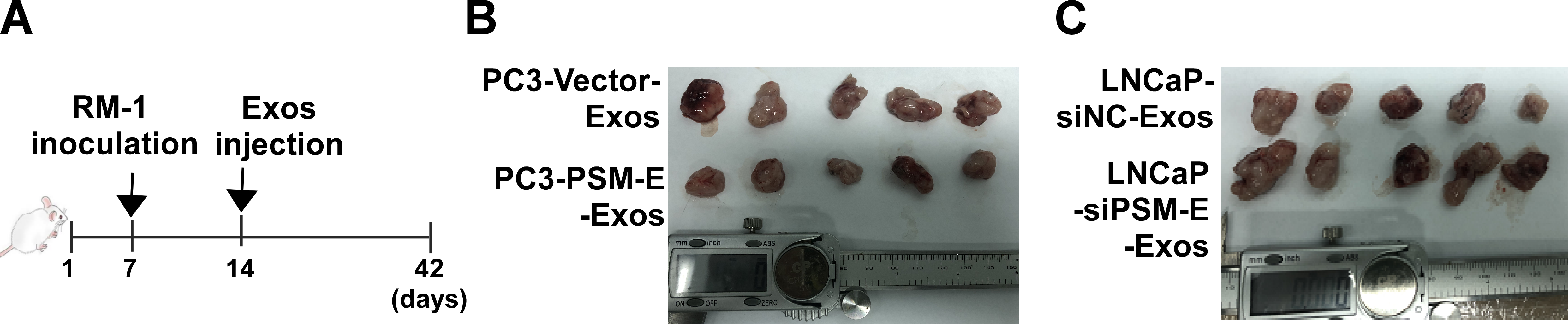

Supplement: Supplementary file 5 — Supplementary Material 5 [file 40364_2024_685_MOESM5_ESM.tif]
