# Supplementary material for: Exosomal PSM-E inhibits macrophage M2 polarization to suppress prostate cancer metastasis through the RACK1 signaling axis
Source: Biomark Res. 2024 Nov 14;12:138. doi: 10.1186/s40364-024-00685-8 (PMC11562865; doi:10.1186/s40364-024-00685-8)
Supplement: Supplementary file 6 — Supplementary Material 6 [file 40364_2024_685_MOESM6_ESM.docx]

**Table S1. The clone primer and RNAi sequences used in this article.**

| **Primer** | |  | | **Sequence 5'-3'** | |
| --- | --- | --- | --- | --- | --- |
| pSin-PSM-E-Flag | Forward | | TGTCGTGAGGAATTGGGATCCGCCACCATGGATTACAAGGATGACGACGATAAGACTGCAGGATCTAGCTATCCATTGTT | | |
|  | Reverse | | ATGCGGATCACTAGTGCTAGCTTAGGCTACTTCACTCAAAGTCTCTGC | | |
| pSin-PSM-E-ΔPA-Flag | Forward | | TGTCGTGAGGaattgGGATCCGCCACCatgGATTACAAGGATGACGACGATAAGAACTTTTCTACACAAAAAGTCAAGAT | | |
| pSin-PSM-E-TFR-Flag | Forward | | TGTCGTGAGGaattgGGATCCGCCACCatgGATTACAAGGATGACGACGATAAGAATTTTACAGAAATTGCTTCCAAGT | | |
| pSin-RACK1-HA | Forward | | TGTCGTGAGGAATTGGGATCCGCCACCATGTACCCATACGACGTCCCAGACTACGCTACTGAGCAGATGACCCTTCGT | | |
|  | Reverse | | ATGCGGATCACTAGTGCTAGCTCAATGGTGATGGTGATGATGGCGTGTGCCAATGGTCACC | | |
| pSin-WD2-7-HA | Forward | | TGTCGTGAGGaattgGGATCCGCCACCatgTACCCATACGACGTCCCAGACTACGCTggtcactcccactttgttagtgat | | |
| pSin-WD3-7-HA | Forward | | TGTCGTGAGGaattgGGATCCGCCACCatgTACCCATACGACGTCCCAGACTACGCTggccataccaaggatgtgctg | | |
| pSin-WD4-7-HA | Forward | | TGTCGTGAGGaattgGGATCCGCCACCatgTACCCATACGACGTCCCAGACTACGCTagccactcagagtgggtgt | | |
| pSin-WD5-7-HA | Forward | | TGTCGTGAGGaattgGGATCCGCCACCatgTACCCATACGACGTCCCAGACTACGCTggccacacaggctatctgaac | | |
|  |  | |  | |  |
| **siRNA** |  | | **Sequence 5'-3'** | | |
| NC siRNA | | | UUCUCCGAACGUGUCACGUTT | | |
| PSM-E siRNA-1 | | | CUGAGAACAUCAAGAAGUU dTdT | | |
| PSM-E siRNA-2 | | | GGCAAAUCUCUUUAUGAAA dTdT | | |
| PSM-E siRNA-3 | | | GCGAUCUAGUGUAUGUUAA dTdT | | |

| **Primer** |  | **Sequence 5'-3'** |
| --- | --- | --- |
| CCL17 | Forward | GAGCCATTCCCCTTAGAAAG |
|  | Reverse | AGGCTTCAAGACCTCTCAAG |
| CCL18 | Forward | CTCCTTGTCCTCGTCTGCAC |
|  | Reverse | GGTTAGGAGGATGACACCTGG |
| CCL22 | Forward | ATTACGTCCGTTACCGTCTG |
|  | Reverse | TAGGCTCTTCATTGGCTCAG |
| iNOS | Forward | CGTGGAGACGGGAAAGAAGT |
|  | Reverse | GACCCCAGGCAAGATTTGGA |
| IL-1β | Forward | GCTGCTTCCAAACCTTTGAC |
|  | Reverse | AGCTTCTCCACAGCCACAAT |
| CD68 | Forward | CGAGCATCATTCTTTCACCAGCT |
|  | Reverse | ATGAGAGGCAGCAAGATGGACC |
| GAPDH | Forward | AGGCCGGTGCTGAGTATGTC |
|  | Reverse | TGCCTGCTTCACCACCTTCT |

**Table S2. The real time RT-PCR primer sequences used in this article.**

**Supplemental Figure Legend**

**Figure S1. Overexpressing or downregulating PSM-E in 293T, PC3 or LNCaP cells.** (**A**) Western blot analysis of PSM-E expression levels in 293T and PC3 cells that transfected with Flag epitope-tagged PSM-E plasmid compared to the vector group. (**B**) Western blot analysis of PSM-E expression levels in LNCaP cells treated with NC and PSM-E siRNA.

**Figure S2 Exosomal PSM-E can be transferred to HFF-1 fibroblast cell line**. (**A**) PC3 and 293T cells transfected with Flag epitope-tagged PSM-E plasmid were co-cultured with HFF-1 cells induced by PMA in a transwell plate. (**B**) PSM-E expression in HFF-1 cells treated with purified exosomes derived from PC3-Vector or PC3-PSM-E-Flag cells.

**Figure S3. Human monocyte THP-1 differentiation into M2-like macrophages.**

(**A**) Real time RT-PCR analysis of the expression of the macrophage maker CD68 was performed. (**B**) Flow diagram showing monocyte differentiation into M2 macrophages. (**C**) Real time RT-PCR analysis of the expression of typical M2 markers (CCL17, CCL18 and CCL22) in PMA-pretreated THP-1 cells that treated with IL4. Unpaired *t*-test was used to analyze two groups of data. ***p* < 0.01, and *** *p* < 0.001.

**Figure S4. Endogenous** **PMS-E interacts with RACK1-HA.** LNCaP cells co-transfected with siRNAs targeting PSM-E and plasmid encoding and HA-tagged RACK1 were used for co-IP assay.

**Figure S5. Exosomal PSM-E restrains tumor growth of PCa *in vivo*. (A)** Schematic diagram of constructing a mouse subcutaneous tumorigenesis model. **(B)** Representative images of subcutaneous tumors in groups treated with purified exosomes derived from PC3-Vector and PC3-PSM-E cells. **(C)** Representative images of homograft tumors in groups treated with purified exosomes derived from LNCaP-siNC and LNCaP-siPSM-E cells.
